# Peripheral Glia Have a Pivotal Role in the Initial Response to Axon Degeneration of Peripheral Sensory Neurons in Zebrafish

**DOI:** 10.1371/journal.pone.0103283

**Published:** 2014-07-24

**Authors:** Holly M. Pope, Mark M. Voigt

**Affiliations:** Department of Pharmacological and Physiological Science, Saint Louis University School of Medicine, St. Louis, Missouri, United States of America; Boston Children’s Hospital and Harvard Medical School, United States of America

## Abstract

Axon degeneration is a feature of many peripheral neuropathies. Understanding the organismal response to this degeneration may aid in identifying new therapeutic targets for treatment. Using a transgenic zebrafish line expressing a bacterial nitroreductase (Ntr)/mCherry fusion protein in the peripheral sensory neurons of the V, VII, IX, and X cranial nerves, we were able to induce and visualize the pathology of axon degeneration *in vivo*. Exposure of 4 days post fertilization Ntr larvae to the prodrug metronidazole (Met), which Ntr metabolizes into cytotoxic metabolites, resulted in dose-dependent cell death and axon degeneration. This was limited to the Ntr-expressing sensory neurons, as neighboring glia and motor axons were unaffected. Cell death was rapid, becoming apparent 3–4 hours after Met treatment, and was followed by phagocytosis of soma and axon debris by cells within the nerves and ganglia beginning at 4–5 hours of exposure. Although neutrophils appear to be activated in response to the degenerating neurons, they did not accumulate at the sites of degeneration. In contrast, macrophages were found to be attracted to the sites of the degenerating axons, where they phagocytosed debris. We demonstrated that peripheral glia are critical for both the phagocytosis and inflammatory response to degenerating neurons: mutants that lack all peripheral glia (*foxD3^−/−^;* Ntr) exhibit a much reduced reaction to axonal degeneration, resulting in a dramatic decrease in the clearance of debris, and impaired macrophage recruitment. Overall, these results show that this zebrafish model of peripheral sensory axon degeneration exhibits many aspects common to peripheral neuropathies and that peripheral glia play an important role in the initial response to this process.

## Introduction

Currently, millions of people suffer from peripheral neuropathies (PN) that arise from numerous causes, including chemotherapeutics, alcohol, toxins, viruses, physical trauma, genetics, and diabetes [1]–[7]. In addition, with the increased incidence of obesity, cases of diabetic neuropathy are on the rise [8]. PNs are characterized by abnormal signaling in the affected nerves [9], which manifests as a variety of symptoms that differ depending on the type of nerve that is damaged. Symptoms can include pain, tingling or numbness in sensory PNs [10], impaired motor ability in motor PNs [11], and autonomic dysfunction in PNs of visceral nerves [12]. Few treatments are available to help alleviate symptoms and there are no cures [2]. Development of relevant models to study aspects of the pathological response in PNs is a critical first step in the design and/or improvement of therapeutics.

Axon degeneration is a feature common to many PNs [13]. With the close association of axons and their ensheathing glia, it would be expected that glia have a pivotal role in the response to axon degeneration. Much of the research on the glial response has focused on myelinating Schwann cells of peripheral nerves, as opposed to non-myelinating glia. These different types of glia associate with axons that differ in their modalities. For example, unmyelinated axons are small fibers that relay signals for pain and temperature, while myelinated axons are larger fibers involved in motor control, pressure sensation, and autonomic functions [14]. Investigations of myelinating Schwann cells, primarily in models utilizing transected nerves, have shown that there is a characteristic reaction of these glia in response to axon degeneration (known as Wallerian degeneration [15], [16]). In this process, Schwann cells phagocytose axonal debris, secrete growth promoting factors, and proliferate to form tracts (bands of Bungner) for regenerating axons to follow [17]. They also down regulate genes for myelin, whose breakdown products contains growth inhibiting molecules [15], as well as undergo dedifferentiation to become immature Schwann cells [17]. In addition to this, Schwann cells recruit immune cells, such as macrophages, to engulf axonal debris and remove growth-inhibitory factors [18]. Together, these events create a local environment conducive to regeneration of axons [19]. A similar progression of events occurs in toxin-induced axon degeneration, referred to as Wallerian-like degeneration [9], [20]. The molecular mechanisms in Wallerian and Wallerian-like degeneration are not well understood, even though the anatomical characteristics that occur in response to axon degeneration are well characterized. While many studies have focused on the myelinating Schwann cell response, little research has been done on how non-myelinating glia respond to axon degeneration. Damage to unmyelinated axons is responsible for many sensory PNs, including painful small fiber neuropathies [21], underscoring the importance of understanding how non-myelinating glia respond to degenerating axons.

Axon degeneration has been studied in several animal models that use physical trauma (nerve transection [22], crush [23], ligation [24]) or a toxic insult (e.g. pyridoxine [25], organophosphorous compounds [9], [26]) to initiate axon degeneration. There are several drawbacks to these models, including: 1) inability to visualize degeneration and the organismal response in real time in a live animal, 2) lack of selectivity for one type of axon over another within a given nerve, and 3) improper cell context where non-nerve tissue is also affected by the insult. Zebrafish offer the potential to be a valuable addition to current animal models for several reasons: embryos/larvae are transparent, allowing *in vivo* visualization of cellular processes in real time; transgenic lines allow selective targeting of a specific cell type; and the availability of a drug-based suicide gene technology that permits conditional and selective ablation of targeted cell populations. This suicide system utilizes a combination of the bacterial enzyme nitroreductase (Ntr) and the prodrug metronidazole (Met) that has been used to model other diseases in zebrafish [27]–[30], such as type 1 diabetes, where Ntr-expressing β cells in the pancreas were ablated with Met treatment [28]. When larvae expressing Ntr are treated with the prodrug Met, the enzyme converts the Met into cytotoxic metabolites which cause DNA damage [31], [32] and increases reactive oxygen species (ROS) [33], [34], leading to selective cell death of Ntr-expressing cells.

In the model described in this report, Ntr is expressed in the peripheral sensory neurons of the V, VII, IX, and X cranial nerves, which consist solely of unmyelinated axons at the developmental stages examined. This expression pattern allows for the selective ablation of peripheral sensory neurons and their axons, while sparing the remaining cell types in a nerve. The response of affected neurons and their axons can be visualized using fluorescent microscopy, as the Ntr protein is fused to the red fluorescent protein mCherry. Using this model, we demonstrated selective ablation of peripheral sensory neurons, induced axon degeneration, and observed the resulting pathophysiological response in live zebrafish larvae using time-lapse microscopy. One major response detected was the creation and subsequent phagocytosis of cell debris. The non-myelinating glia found in the ganglia and ensheathing the axons were found to have an important role in both debris phagocytosis and macrophage recruitment, as mutants that lack all peripheral glia had alterations in the clearance of axonal debris as well as impaired recruitment of these immune cells compared to wild type Ntr fish. The glial and inflammatory cell responses to degenerating axons observed in this model resembled those seen in many other studies of axon degeneration [15], [18]. This novel system offers a number of advantages for investigating sensory axon degeneration while sharing many of the same characteristics seen in a large number of PNs caused by toxic or metabolic insults. This zebrafish model can be used for further studies aimed at elucidating the molecular mechanisms of Wallerian-like degeneration and the inflammatory response to axon degeneration, processes that have important clinical relevance.

## Materials and Methods

### Ethics Statement

All experiments were carried out in accordance with the National Institutes of Health Guide for the Care and Use of Laboratory Animals. The Saint Louis University Animal Care Committee approved the procedures used (protocol # 1619) and all efforts were made to minimize the number of animals used and their suffering.

### Maintenance of fish and fish strains

All animal husbandry was carried out as described by Westerfield [35], and staging was carried out using the criteria of Kimmel et al. [36]. For some experiments, reduction of pigmentation of embryos/larvae was achieved by growing embryos in fish water containing phenylthiourea (PTU) at a concentration of 0.0015%. The following strains were used: *Tg(isl:gfp)* [37], *Tg(p2rx3.2(−4.0):gal4vp16)* [38], *Tg(UAS:nsfB-mcherry)* [28], *foxd3^zdf10^* [39], *panther* (gift from Steve Johnson) [40], *Tg(mpx:gfp)* (gift from S. Renshaw, Sheffield Univ.) [41] and Tg(mpeg1:gfp^gl22Tg^) [42] from ZIRC. In *Tg(p2rx3.2(−4.0):gal4vp16);Tg(UAS:nsfB-mcherry)* embryos/larvae, in addition to expression in sensory neurons, there was also variable ectopic expression between individuals within a single clutch. Therefore, larvae were put into separate groups based on Ntr expression, so that individuals with equivalent expression could be compared for a given experiment.

### Metronidazole treatment

Larvae at 4 dpf were treated with metronidazole (Sigma, St. Louis, MO) at a final concentration of 1, 5, or 10 mM in fish water. We found 10 mM to give the maximum degree of ablation, so we used this concentration for our studies. Control fish were treated with fish water alone.

### Epifluorescent Microscopy

Larvae were anesthetized with 0.02% tricaine in fish water and transferred to a 96-well plate. Epifluorescent images were taken using a Nikon TE200 inverted microscope equipped with a CoolSNAP HQ digital camera. MetaMorph software (Universal Imaging Corp) was used to acquire and process images. Cropping and rotating of images was carried out using Adobe Photoshop. For time lapse imaging, larvae were embedded in 0.5% low-melting point agarose containing 0.02% tricaine, and images were taken every 5 minutes.

### Confocal imaging

Live embryos/larvae were anesthetized with tricaine (0.01%) and placed in low-melting point agarose (0.5–1.0%) wells present on specially designed tissue culture dishes: these were manufactured by creating a 10 mm hole in the bottom dish and then covering this hole by gluing a glass cover slip on the external surface of the dish. Imaging was performed using a 20x water immersion objective mounted on an Olympus FV-1000 MPE confocal microscope. All images were handled using either Olympus Fluoview or ImageJ software, with final adjustments to contrast and brightness made using Adobe Photoshop.

### Transmission Electron Microscopy

Larvae at 4 or 5 dpf were anesthetized with 0.02% tricaine in fish water and heads were removed with a small scalpel under a dissecting microscope. Microwave assisted larval fixation and embedding were performed as described [43] using a PELCO BioWave Pro Microwave System coupled to a PELCO Steady Temp water bath (Ted Pella, Redding, CA) with the following modifications: secondary fixation with 1% osmium in 0.1 M sodium cacodylate buffer with 2% sucrose for 30 min RT; washed with distilled water twice for 5 min; dehydrated with 30% EtOH twice for 5 min, 50% EtOH twice for 5 min, 75% EtOH twice for 5 min, 95% EtOH twice for 5 min, 100% EtOH twice for 5 min, 100% propylene oxide twice for 5 min; infiltrated with propylene oxide:resin (1∶1) 20 min followed by 100% resin for 30 min. Transverse thick sections (0.5 µm) were stained with Toluidine Blue before thin sections were taken and stained on grids. The Reconstruct program [44], which aligns serial sections to give a 3-dimensional view of structures of interest was used as an aid in verifying that we were examining the correct ganglia and axons in the larvae. Using Toluidine Blue sections from a control Ntr larva, we took serial transverse sections every 5 um from the region posterior to the ear. We then outlined the putative ganglia and axons, and Reconstruct made a 3D model of these structures (data not shown). The resulting structures mimicked the shapes and sizes of the ganglia and axons seen using fluorescence microscopy. This reference fish was used to determine the proper location of axons and ganglia is subsequent sectioning of other larvae. Multiple TEM images of a single structure that spanned a large area were stitched together using Microsoft Image Composite Editor. Quantitation of TEM images to obtain the area of structures was performed using the ROI Manager Tools plug-in for ImageJ.

### Acridine Orange stain for apoptosis

Larvae at 4 dpf were treated with control PTU water or 10 mM Met for the indicated times, then live larvae were incubated in 10 ug/mL acridine orange in fish water for 20 min in the dark at RT. Larvae were then rinsed three times with fish water, anesthetized with tricaine, and put in a 96-well plate for imaging [45]. Acridine orange stained cells located in the ganglia were counted using the ITCN (Image-based Tool for Counting Nuclei) plugin for Image J [46]. The detection-limit threshold for AO stained cells was set using a single image, and this threshold was kept for all subsequent image analyses.

### Sudan Black stain for neutrophils

Larvae at 4 dpf were treated with control PTU water or 10 mM Met for the indicated times, fixed in 4% paraformaldehyde for either 2 hr RT or overnight at 4°C, washed twice for 5 min in PBS, then stained with Sudan Black (Sigma, St. Louis, MO) for 20 min. Larvae were washed in 70% EtOH until non-specific staining disappears, then rehydrated with a series of EtOH dilutions in PBS plus 0.1% Tween 20 [47].

### Neutral Red vital stain for macrophages

Larvae at 4 dpf were treated with control PTU water or 10 mM Met for the indicated times, stained by incubating in 2.5 ug/mL Neutral Red solution (Sigma, St. Louis, MO) in PTU water for 6–8 hr in the dark, then rinsed with PTU water and kept in PTU water ON to wash out background staining [48]. Larvae were imaged at 5 dpf.

### Neutrophil Tracking

Multiple time lapse videos of 4 dpf *Tg(mpx:gfp)/Tg(p2rx3.2(−4.0):gal4vp16/UAS:nsfB-mcherry)* larvae treated with control PTU water or 10 mM Met were taken every 30 sec for 30 min. Together, these videos spanned a treatment time of 6 hr. The ImageJ plugin MTrackJ [49] was used to track and calculate movement parameters of GFP-expressing cells.

### Cell Counting and Statistics

Sudan Black and Neutral Red stained larvae were embedded on their side in 1% low melt point agarose on an imaging plate and the focus was adjusted to obtain serial images from consecutive planes in the larvae. Using Adobe Photoshop, stained cells were marked in each image and the markings were overlaid to ensure that each cell was only counted once. Marked cells were counted manually in a region that measured a half ear-length from all sides of the ear. Students t test was used to compare means.

## Results

### Metronidazole selectively killed Ntr-expressing cells causing axon degeneration in a dose dependent manner

**Figure 1:**
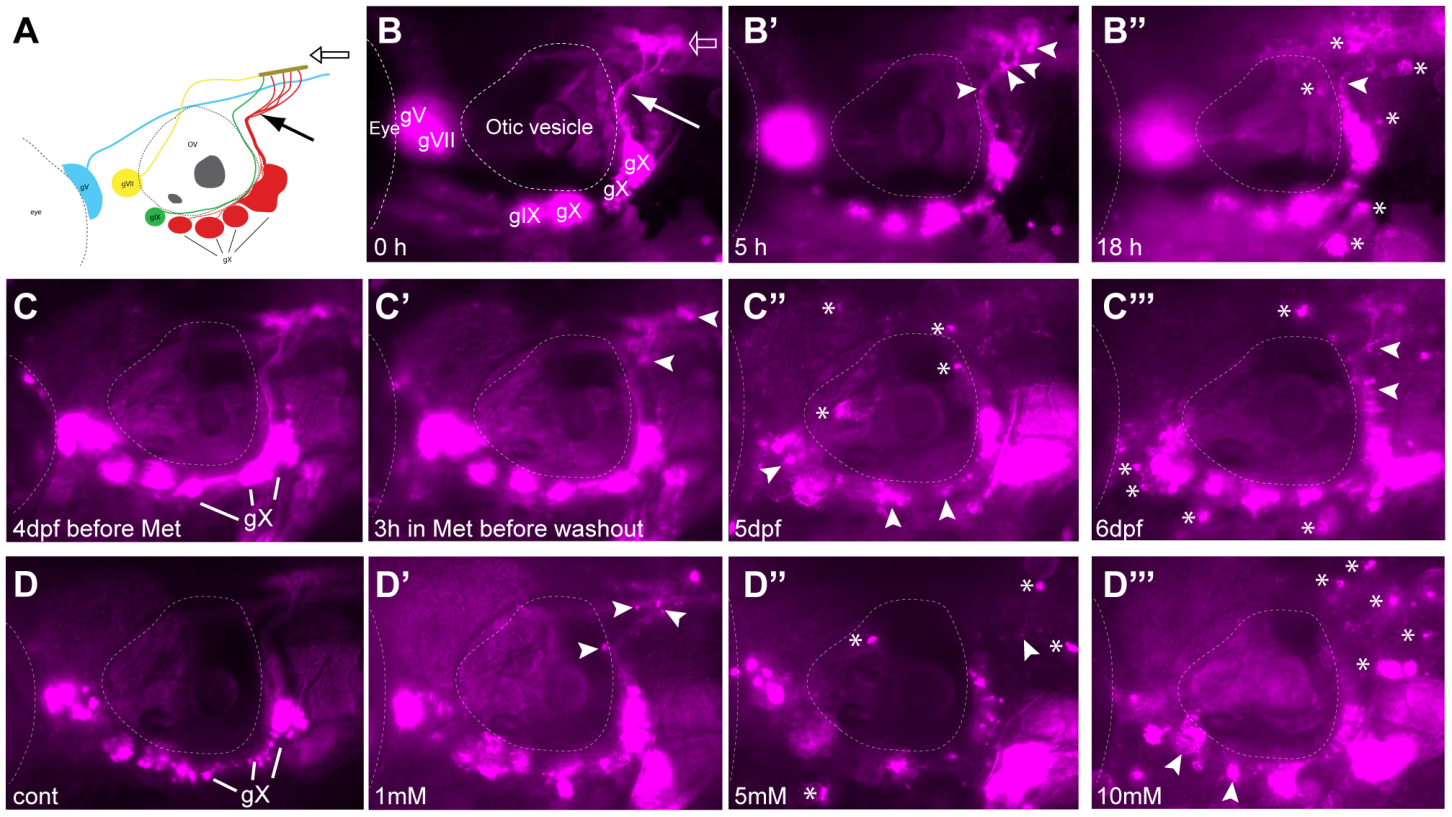
Metronidazole treatment in 4 days post fertilization (dpf) Ntr larvae. **A)** Diagram of expression pattern of *p2rx3.2(−4.0):gal4vp16;UAS:nsfB-mcherry* (referred to as Ntr). Projection of axons indicated with closed arrow. Terminal field in hindbrain (plexus) labeled with open arrow. B) Epifluorescent image of 4 dpf Ntr with labeling as in A. Projections of axons indicated with closed arrow; plexus indicated with open arrow. Select images from a time lapse video of 4 dpf Ntr in 10 mM Met at **B)** 0 hr, **B’)** 5 hr, and **B’’)** 18 hr. Puncta along axons (arrowheads) was seen at 5 hrs. By 18 hrs, motile cells containing cellular debris (asterisks) were seen around the ganglia and axons. **C)** 4 dpf Ntr imaged before Met treatment and **C’)** after 3 hrs in 10 mM Met (arrowheads point to puncta in axons). Met was then washed out of the larva, followed by imaging at **C’’)** 5 dpf and **C’’’)** 6 dpf (arrowheads point to degenerating ganglia and axons; asterisks indicate red motile cells containing debris). Same fish imaged at each time point. **D)** Dose response of 4 dpf Ntr given fish water (control vagal ganglia labeled gX), **D’)** 1 mM, **D’’)** 5 mM, or **D’’’)** 10 mM Met overnight for 18 hrs, followed by imaging at 5 dpf. Arrowheads point to degenerating ganglia and axons; asterisks indicate debris. Anterior is to the left; dorsal is at the top. Eye and otic vesicle (OV) are outlined. Cranial sensory ganglia labeled gV, gVII, gIX, and gX in panels A,B; vagal ganglia labeled gX in panels C,D.

We have developed a zebrafish model of peripheral sensory axon degeneration, exhibiting characteristics similar to those seen in many sensory PNs, utilizing the nitroreductase/metronidazole (Ntr/Met) suicide system [29], [30]. The transgenic line Tg(*p2rx3.2:gal4vp16;UAS:nsfB-mcherry*) (from here on abbreviated as Ntr) [29] provides variegated expression of the E. *coli* nitroreductase (*nsfB*) protein, fused to mCherry, in the peripheral sensory neurons contributing to the V, VII, IX, and X cranial nerves. It is important to note that due to the variegated expression pattern, only a subpopulation of sensory neurons are targeted. This lack of complete ablation mimics many PNs in which only a subpopulation of sensory axons is affected [50], [51]. An added advantage in using this chimeric protein is that it permits the visualization of not only the neuronal somas, but their axonal extensions as well: Figure 1A–B shows the typical expression pattern of 4 days post fertilization (dpf) Ntr larvae.

Experiments using video time lapse analysis of 4 dpf Ntr larvae treated with 10 mM Met over a 20 hour period (Figure 1B–B’’) suggested that Ntr+ axons exhibited a degenerative phenotype: they showed a progressive diffuse appearance, presumably due to loss of membrane integrity and subsequent engulfment by phagocytic cells present within the nerve. Distinct fluorescent puncta could be seen forming along the peripheral axons and in the plexus (the region where the sensory axons arborize within the hindbrain) starting at 2–3 hours post exposure (arrowheads Figure 1B’, Movie S1). This was followed 1–3 hours later by the appearance of motile cells that began to accumulate mCherry as they moved into and out of the nerve (referred to as red-labeled motile cells) (asterisks, Figure 1B”). This motile-cell labeling is presumed to arise from the phagocytosis of debris from Ntr/mCherry-containing axons and somas. The labeled motile cells persisted for up to 2 days after removal of the Met (asterisks in Figure 1C–C”’). At this dosage of Met, it appeared that there was a complete loss of intact mCherry^+^ axons after overnight exposure, demonstrating the high efficiency of this drug in ablating peripheral sensory neurons expressing the Ntr protein (Figure S1). The effects of Met were dose-dependent as exposure to lower concentrations of Met (1 and 5 mM) for 18 hours yielded less puncta and fewer motile cells, with less apparent damage to labeled axons (Figure 1D–D’’’). These results were never seen in untreated Ntr or Met-treated wild-type (non-Ntr expressing) larvae.

**Figure 2:**
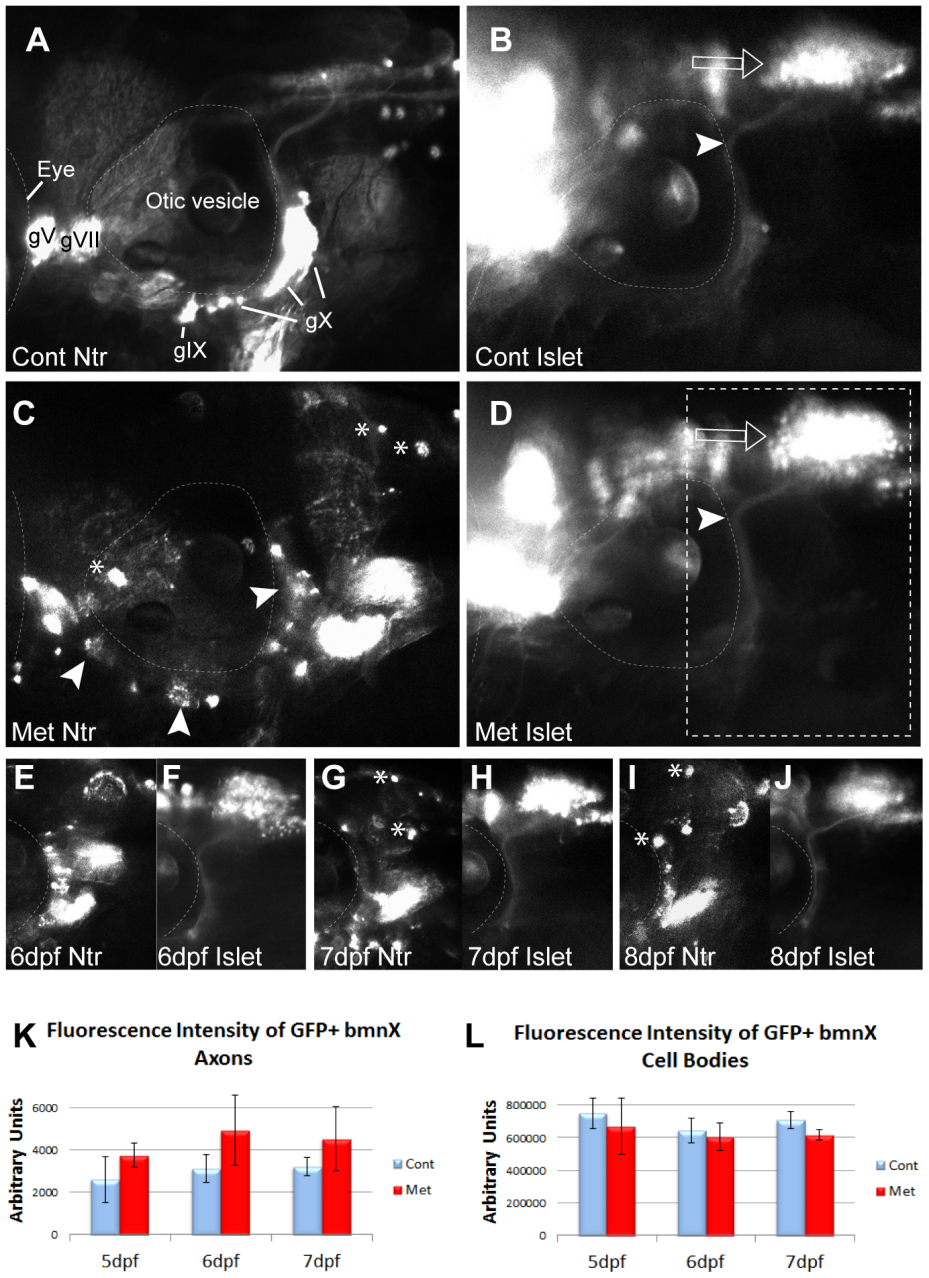
Metronidazole does not have a bystander effect. **A,B)** 4:gfp;Ntr incubated in fish water (control) for 18 h and imaged at 5 dpf. A) Ntr-expressing sensory neurons. B) Isl1:gfp-expressing branchiomotor neurons (bmn). Arrowhead indicates axons; open arrow indicates motor cell bodies in hindbrain. **C,D)** 4 dpf Isl1:gfp;Ntr treated with 10 mM Met for 18 h and imaged at 5 dpf. C) Ntr-expressing sensory neurons/axons degenerate. Arrowheads point to degenerating cell bodies; asterisks indicate mCherry-containing debris. D) Isl1:gfp-expressing bmn remain intact while sensory neurons contributing to the same nerve degenerate. Open arrow points to motor neuron cell bodies; arrowhead indicates motor axons. Outlined box indicates region of focus in E–J. **E–J)** 4 dpf Islet:gfp;Ntr kept in 10 mM Met and imaged for 3 consecutive days. **E)** 6 dpf Ntr; **F)** 6 dpf Isl1:gfp; **G)** 7 dpf Ntr; **H)** 7 dpf Isl1:gfp; **I)** 8 dpf Ntr; **J)** 8 dpf Isl1:gfp. Water changes containing fresh Met were performed each day after imaging. Asterisks indicate some of the debris containing mCherry seen after Met-treatment. Met had no significant effect on Islet-expressing bmn, while Ntr-expressing sensory neurons contributing to the same nerve degenerated. K,L) Quantitation of fluorescence intensity of isl1:gfp bmn axons (K) and cell bodies (L) from individual larvae maintained in 10 mM Met for 3 days showed no change in fluorescence during peripheral sensory axon degeneration. Fluorescence intensity was quantitated using ImageJ, with the same region of interest used for each analysis. Integrated density, area, mean and background fluorescence were measured and from these values, corrected total cell fluorescence (given in arbitrary units) was calculated (n = 3 larvae/treatment group) and analyzed (t-test). Eye is outlined in A–D, otic vesicle outlined in all panels. Cranial ganglia labeled gV, gVII, gIX and gX. Anterior to left, dorsal at top for all panels.

The affected V, VII, IX, and X cranial nerves are mixed, containing motor as well as sensory axons. To determine if there was a bystander effect (cytotoxic metabolites created during Met treatment of Ntr-expressing sensory axons released into the extracellular environment and affecting the neighboring Ntr-negative motor axons), we crossed Ntr fish to a transgenic line that expresses GFP in branchiomotor neurons (*Tg(islet:gfp))* [37]. This allowed us to visualize both sensory (red) and motor (green) axons in the same fish (Figure 2). When 4 dpf *Tg(islet:gfp);Ntr* larvae were treated with 10 mM Met overnight, the Ntr-expressing sensory axons appeared punctate, red motile cells were seen in the surrounding tissue, and the ganglia showed signs of degeneration (Figure 2C). The GFP-expressing motor axons located in this same nerve appeared intact, with no GFP seen in the surrounding tissue and no decrease in GFP fluorescence after overnight treatment (Figure 2D, K). The somas of the motor neurons, located in the hindbrain, appeared intact and no change was detected in the overall level of fluorescence in the branchiomotor X nuclei (open arrows, Figure 2B,D,L). When these larvae were kept in 10 mM Met for up to 8 days, the Ntr-expressing sensory axons had completely degenerated (Figure 2E,G,I) and yet Met treatment did not have an effect on motor axons (Figure 2F,H,J,K). Together, these data demonstrate that there is no discernible bystander effect arising from either Met treatment or Met metabolism by Ntr-expressing cells.

**Figure 3:**
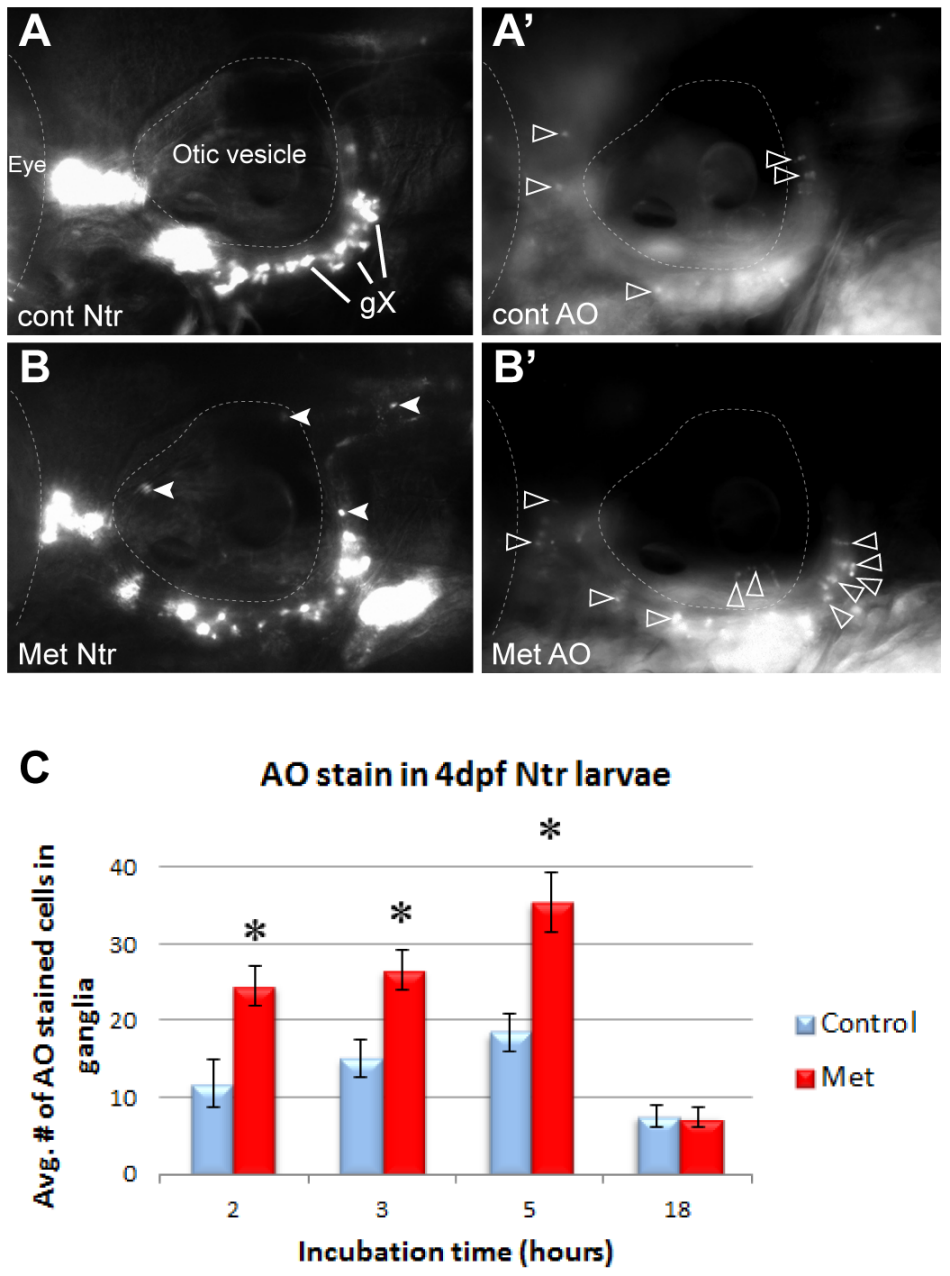
Metronidazole caused neuronal cell death in Ntr-expressing cells. **A)** 4 dpf Ntr incubated in fish water (control) for 5 hr, followed by **A’)** staining and imaging with Acridine Orange (AO). AO-stained cells indicated with open arrowheads. **B)** 4 dpf Ntr treated with 10 mM Met for 5 hr (closed arrowheads indicate puncta in axons and debris containing mCherry), followed by **B’)** AO staining and imaging. Open arrowheads indicate AO stained cells. **C)** 4 dpf Ntr treated with 10 mM Met for various times, followed by AO staining, imaging, and counting in the ganglia. Graph indicates the average number of AO-stained cells in the ganglia for each treatment group at 2 h (control 11.75±3.14 cells, n = 12 and Met 24.54±2.67 cells, n = 24, p<0.05), 3 h (control 15.05±2.35 cells, n = 20 and Met 26.55±2.61 cells, n = 29, p<0.05), 5 h (control 18.44±2.35 cells, n = 16 and Met 35.48±3.85 cells, n = 24, p<0.05), and 18 h (control 7.54±1.4 cells, n = 21 and Met 7.46±1.22 cells, n = 21, ns). There was a significant increase in cell death during the first few hours in Met (2–5 hr), but not after 18 hr. ns, not significant.. For each image, anterior is to the left; dorsal is at the top. Eye and otic vesicle (OV) are outlined, vagal ganglia labeled gX in panel A.

To validate that Met treatment resulted in the death of Ntr-expressing peripheral sensory neurons, the vital stain (Acridine Orange (AO)) was used to label dead and dying cells [52]. We treated 4 dpf Ntr larvae with control PTU water or 10 mM Met for 2, 3, 5, or 18 hr, followed by AO staining and counting of labeled cells located in the branchial ganglia (Figure 3). In non-Met treated larvae, some AO staining was detectable (Figure 3A,A’). However, AO staining was increased in larvae treated with Met (Figure 3B,B’). Quantitation revealed significant increases in cell death after a 2, 3, or 5 hr exposure to Met, but not after overnight treatment (Figure 3C). These findings indicated that Met treatment did result in cell death in Ntr-expressing cells and that the majority of cell death occurred 2–5 hours into Met treatment.

Together, these experiments confirmed that selective ablation of peripheral sensory neurons and their axons could be visualized in real time using the Ntr/Met system in zebrafish.

### Transmission electron microscopy showed the effects of Met treatment in Ntr larvae

**Figure 4:**
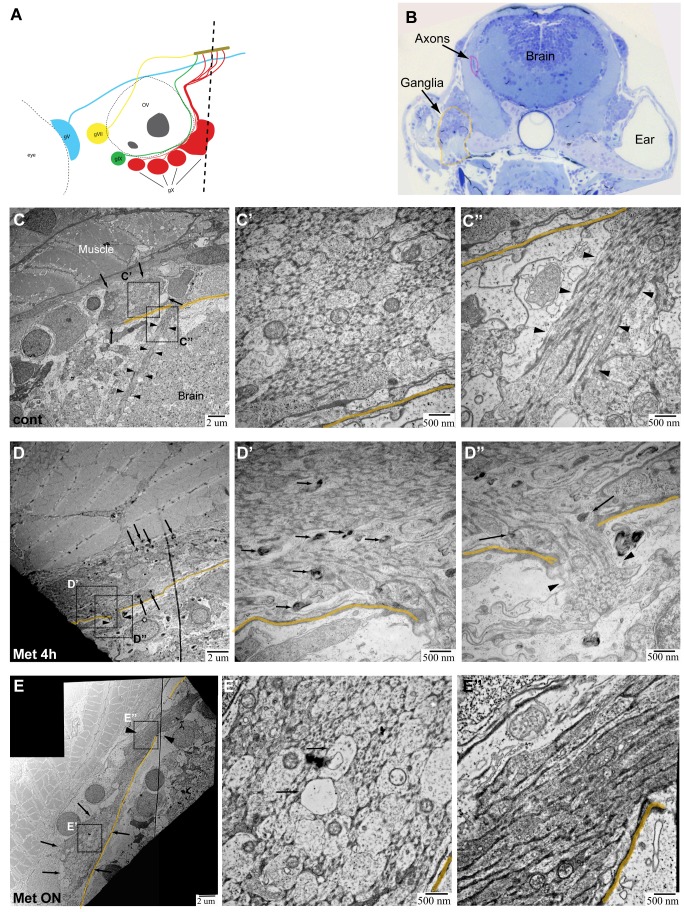
TEM images of axons in Ntr larvae. **A)** Diagram of ganglia and axons. Dotted line indicates location of transverse sections in 4 or 5 dpf larvae. **B)** Representative Toluidine Blue (TB) stained transverse section shows location of axons (magenta) and ganglia (orange) in 5 dpf larvae. **C,C’,C’’)** 5 dpf untreated Ntr. C) Cross section of axon bundle (arrows) and longitudinal view of axons (arrowheads) entering hindbrain (6000x). Boundary between brain and periphery (basement membrane) indicated in orange in all images. C’) Cross section of axons (30,000x). Small and medium diameter axons are visible. C’’) Longitudinal view of parallel axons entering brain (arrowheads) (30,000x). **D,D’,D’’)** 4 dpf Ntr treated with 10 mM Met for 4 h. D) Cross section of axon bundle and longitudinal view of axons (arrowheads) entering hindbrain (7500x). Electron-dense puncta (arrows) are seen at this magnification. D’) Cross section of axon bundle (30,000x). Damaged axons containing dark puncta (arrows). D’’) Axons entering brain (arrowheads) show dark puncta (arrows) (30,000x). **E,E’,E’’)** Ntr at 5 dpf after 18 hr-treatment with 10 mM Met. E) Cross section of axon bundle (arrows) and longitudinal view of axons (arrowheads) entering hindbrain (7500x). E’) Cross section of axon bundle (30,000x). Individual axons (arrows) appear empty and have few microtubules and neurofilaments. E’’) Axons entering brain (30,000x) appear darker and are more electron-dense than control axons (compare to C’’).

Epifluorescence imaging and AO staining provided strong evidence that axon degeneration and cell death were the result of Met treatment in Ntr larvae. However, these techniques are indirect indicators. Therefore, we wanted to directly verify that these processes did occur by examining tissues at an ultrastructural level using transmission electron microscopy (TEM). 4 dpf Ntr larvae were treated with fish water or 10 mM Met for 4 or 18 hours, then fixed and processed for TEM. Transverse sections of larvae were taken posterior to the ear (Figure 4A) and stained with Toluidine Blue before being sectioned for TEM. Figure 4B is a representative Toluidine Blue stained section from an untreated larva showing the location of the two areas chosen for high resolution imaging: peripheral axons of the vagus nerve near the transition zone of the hindbrain, the area between the brain and the periphery where the axons cross the boundary of the CNS, and the caudal-most vagal ganglion.

TEM images of the transition zone showed a cross section of the vagal nerve bundle in untreated control (Figure 4C), 4 h Met-treated (Figure 4D), and overnight Met-treated (Figure 4E) larvae. In control larvae, this nerve cross section displayed several small-diameter and some medium-diameter axons containing mitochondria (Figure 4C’). Interestingly, there was no evidence of myelinated axons in the nerve at this age, an observation in keeping with previous work reporting the lack of myelin basic protein (mbp) expression in the cranial nerves up to 11dpf [39]. Also in control larvae, the longitudinal view of axons showed them projecting as a uniform bundle (arrowheads) into the hindbrain (Figure 4C’’). Previous work from our group has shown that the vagal nerve consists of two bundles of axons, one motor and the other sensory, that exist in a side-by-side configuration and are not intertwined [53]; however, in these TEM images it was not possible to distinguish which population was motor and which was sensory. Common features of axon degeneration seen with TEM analysis are dark puncta located within axons, disrupted microtubules, the presence of vacuoles within an axon bundle, as well as mitochondrial damage [13], [54]–[56]. In the nerve cross section of 4 h Met-treated larvae, dark puncta were seen in some axons (arrows, Figure 4D’), while in overnight Met-treated larvae, there was an apparent increase in the number of axons with sparse microtubules, appearing as vacuole-like structures (arrows, Figure 4E’). These structures were seen alongside normal-looking axons (Figure 4E’), which are most likely motor axons and non-Ntr expressing sensory axons. Also with Met treatment, damaged mitochondria were observed within axons (Figure S2). Quantitation of mitochondria in Ntr larvae showed that the percentage of abnormal mitochondria significantly increased from 30.8% (±8.9%, n = 82 mitochondria) in untreated controls to 71.8% (±14.1%, n = 52 mitochondria) in 4 hour Met-treated and to 70.5% (±9.8%, n = 88 mitochondria) in18 hour Met-treated larvae (Figure S2C). Another sign of unhealthy axons is an increase in electron density [57], [58] - this feature was more prominent in overnight Met-treated larvae when compared to control or 4 hour Met-treated Ntr larvae (compare Figure 4E’’ with C’’,D’’). Quantitation of overall axon damage in Ntr larvae showed that the percent of damaged axons from a nerve cross section significantly increased from 16.2% (±2.8%, n = 10) in untreated larvae to 39.9% (±6.1%, n = 5) after a 4 hour Met treatment (p<0.01). The damaged axons seen in untreated larvae can be attributed to normal ongoing apoptosis of developing neurons and/or fixation artifact. While both of these phenomena could also contribute to the damaged axons seen in Met-treated larvae, the fact that the difference was significant demonstrates that Met treatment did result in increased axon damage.

**Figure 5:**
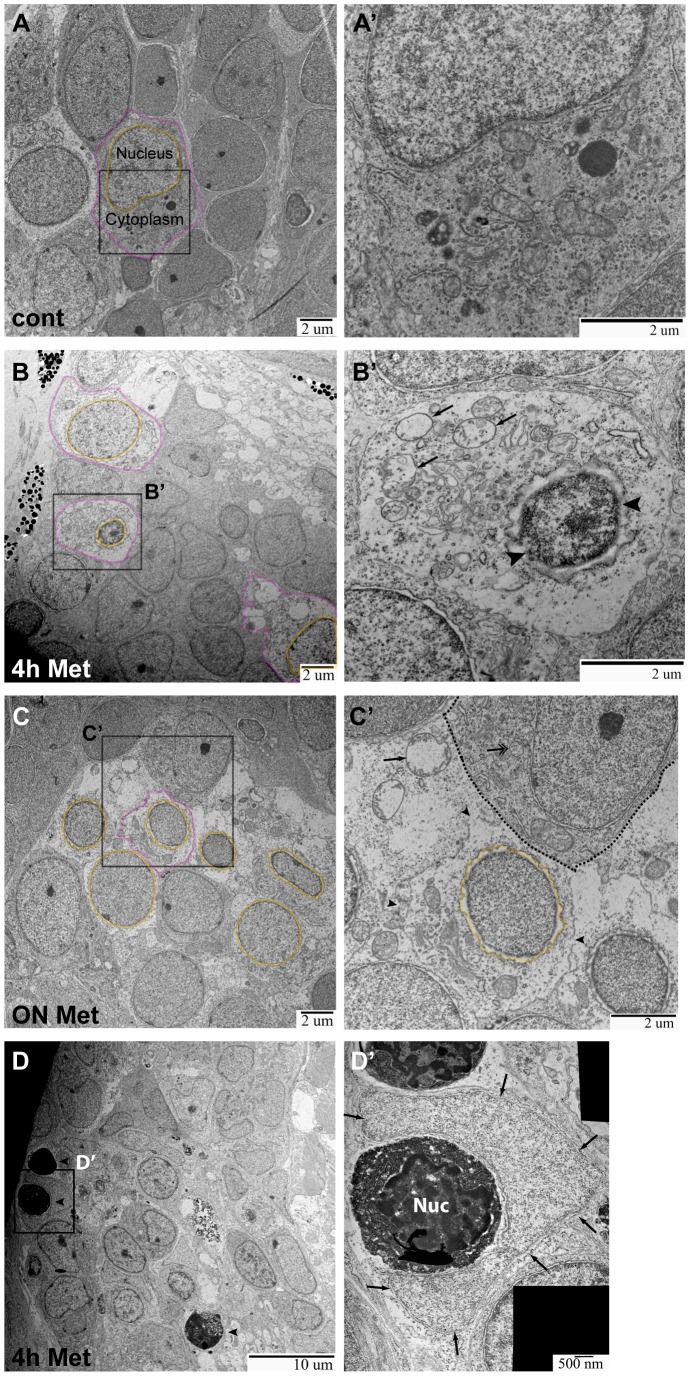
TEM images of ganglia in Ntr larvae. **A,A’)** Ganglia in 5) Individual cell indicated with nucleus (orange) and cytoplasm (magenta) outlined (7500x). A’) Higher magnification of A (15,000x) shows a normal cell. **B,B’)** Ganglia in 4 dpf Ntr treated with 10 mM Met for 4 h. B) Degenerating cell bodies (nuclei in orange; cytoplasm in magenta) are apparent at this magnification (7500x). B’) Higher magnification of B (15,000x) shows one of these damaged cells. The nucleus (arrowheads) has shrunk and organelles in the cytoplasm (arrows) appear damaged. **C,C’)** Ntr at 5 dpf after 18 hr- treatment with10 mM Met. C) Damaged cells lacking discreet cell membranes (nuclei in orange) are apparent at this magnification (7500x). Individual cell outlined in magenta. C’) Higher magnification image of C (15,000x) shows one of these damaged cells. The nucleus (orange) appears ruffled and the cell membrane is broken (arrowheads) and irregular. A neighboring healthy cell is indicated (dashed line) containing a normal organelle (double arrow). For comparison, a swollen organelle from a degenerated cell is indicated with an arrow. **D,D’)** Ganglia in 4 dpf Ntr treated with 10 mM Met for 4 h. D) Dying cells (arrowheads) are apparent at this magnification (4000x). D’) Higher magnification of D (30,000x) shows a dying cell (electron-dense cell body with nucleus (Nuc) being engulfed by a phagocytic cell (arrows).

TEM images of the vagal ganglia allowed detection of both neuron and satellite glia cell bodies (Figure 5). In control larvae, nearly all neuronal cell bodies seen were intact (Figure 5A,A’). In 4 h Met-treated larvae, degenerating cell bodies containing shrunken nuclei (orange) and disorganized cytoplasm (magenta) were observed (Figure 5B,B’) and many of the cellular plasma membranes remained intact. In larvae treated with Met for 18 hours, low magnification revealed that the cytoplasm of neuronal cell bodies appeared disorganized, without discrete organelle membranes, and contained more diffuse white space than in controls (Figure 5C). At a higher magnification, degenerating cell bodies, cell debris, and non-intact membranes were found (Figure 5C’). Quantitation of damage in the ganglion showed that the percent of abnormal cell bodies significantly increased from 38.3% (±6.5%, n = 5) in untreated to 60.8% (±2.0%, n = 5) in 4 hour Met-treated to 85.1% (±14.9%, n = 2) in overnight Met-treated larvae (p<0.05), demonstrating that Met treatment did indeed result in increased neuronal death in the ganglion. That damaged cell bodies were seen in untreated larvae can be attributed to the normal apoptosis of neurons that occurs during development and/or to fixation artifact. The neuronal cell bodies unaffected by Met treatment were most likely not expressing the Ntr transgene. Our TEM analysis revealed signs of degeneration in the neuronal cell bodies of Met-treated Ntr larvae consistent with previous reports that showed neuronal membrane disruption in a toxin-induced axon degeneration mouse model, and with those from a drosophila mutant with disrupted axonal transport leading to axon degeneration [58], [59]. Thus, the results from these TEM studies support the observations made at a macro level with epifluorescence microscopy.

In addition to the changes in neuronal integrity, the TEM images also revealed phagocytic cells wrapped around presumed dead or dying neurons (electron-dense cell bodies) in the ganglia of Met-treated Ntr fish (individual cell indicated with arrows, Figure 5D,D’); these phagocytic cells were not seen in control larvae. There are several types of phagocytic cells that could be engulfing neuronal debris, including satellite glia, neutrophils and macrophages. It was not possible to distinguish the cell type(s) carrying out phagocytosis using TEM. Therefore, our next sets of experiments were designed to investigate whether neutrophils and/or macrophages were recruited to the degenerating somas and axons.

### Neutrophils reacted to degenerating neurons

Neutrophils are a type of phagocytic immune cell known to be recruited early in the inflammatory response to injured myelinated nerves [60]. We wanted to test if neutrophils were recruited to degenerating axons/somas in our model and if they comprised a portion of the phagocytic red motile cells seen in the previously described time-lapse videos. 4 dpf larvae were treated with or without Met for various lengths of time, and neutrophils were stained using Sudan black (SB), a dye that stains the lipophilic granules in polymorphonuclear cells [61], and labeled cells in the region of the ganglia and axons were counted. While there appeared to be an increase in SB stained cells in Met-treated larvae, there in fact was not a statistically significant increase between control and Met-treated larvae at any time point tested (Figure S3).

One drawback of SB staining is that it only provides a static snapshot of the number and positions of neutrophils at any given time. To determine if neutrophils were transiently moving through the region of the ganglia and axons, we sought to carry out time-lapse videos of neutrophil movement in response to degenerating axons. To visualize neutrophils, we crossed Ntr fish to a transgenic line that expresses GFP under the control of the promoter of the myeloperoxidase gene *(Tg(mpx:gfp*)) [41], which is a known marker of neutrophils. In these larvae, time-lapse imaging allowed tracking of individual neutrophils (Movies S2 and S3). Using the MTrackJ plugin in ImageJ, various parameters of neutrophil movement were calculated over a 6 hour period [49]. While some neutrophils moved towards, and others away, from the degenerating axons and ganglia, there were neutrophils that were confined to a small area for the duration of the time lapse. These types of movements were seen in both control and Met-treated larvae. While there did not appear to be significant changes in the overall direction of neutrophil movement in response to Met-induced neuron degeneration, there were significant differences in the velocity of their movement (Figure S3): the neutrophils in control larvae maintained a relatively constant velocity over the 6 hrs, while neutrophil velocity in Met-treated larvae fluctuated between greater than and equal to those in untreated larvae. This type of movement is characteristic of chemokinesis, which is a stimulated non-directional movement shown by activated neutrophils [62], [63].

### Macrophages were recruited to degenerating neurons

**Figure 6:**
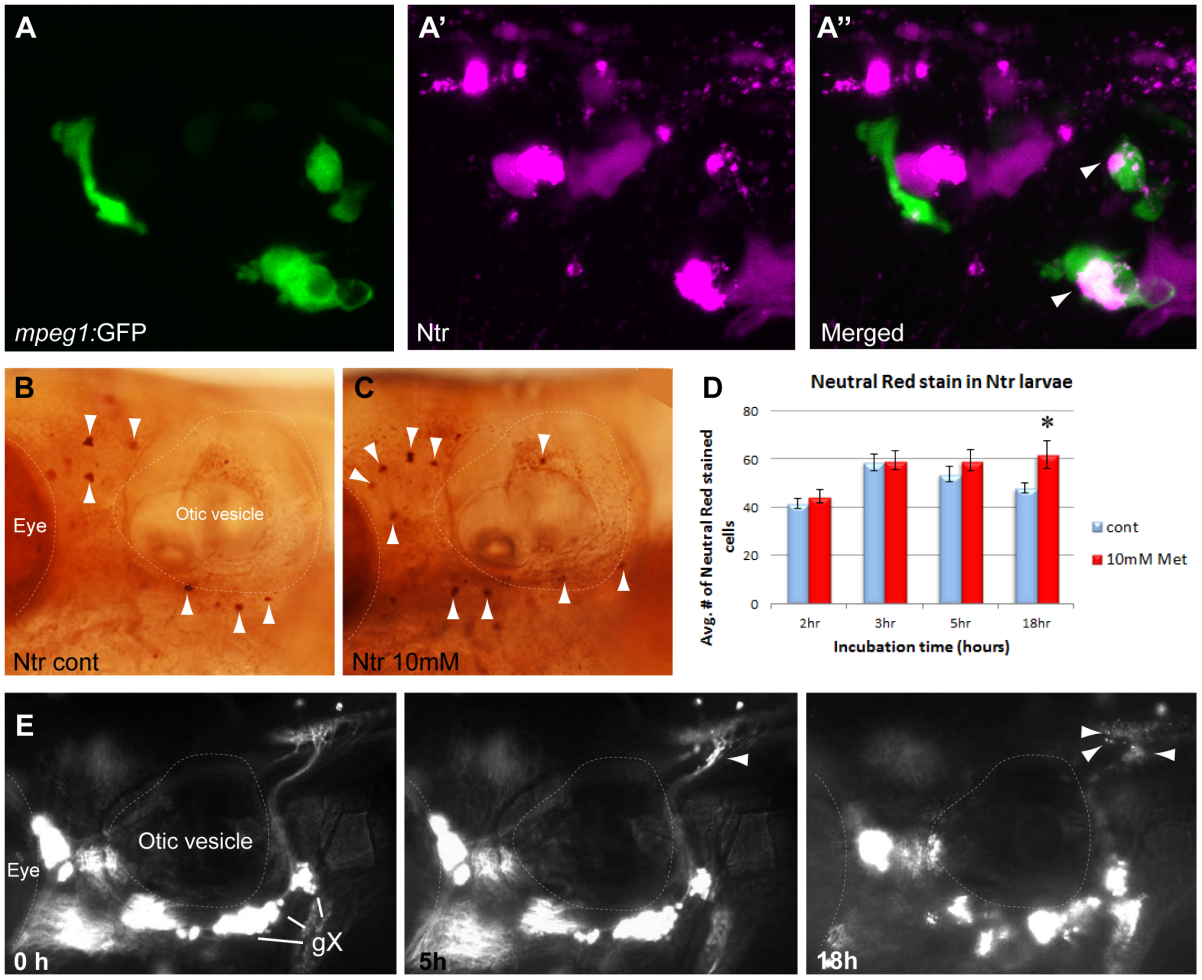
Macrophage response to degenerating neurons. **A,A’,A”)** 5:gfp;Ntr larvae after a 24 hr treatment with 10 mM Met. **A)** Green channels shows macrophages located in a gX subganglion. **A’)** Magenta channel shows degenerating cell bodies. **A”)** Merged image shows colocalization (white/pink) of debris with macrophages. **B,C)** Neutral Red (NR) stained macrophages (arrowheads) in B) untreated or C) 3 hr Met-treated Ntr larvae at 4 dpf. **D)** Quantitation of NR-stained cells in 4 dpf Ntr treated with fish water (control) or 10 mM Met for various times. Graph shows average number of NR-stained cells per treatment group at 2 h (control 41.53±2.13 cells, n = 15 and Met 44.65±2.82 cells, n = 17, ns), 3 h (control 58.7±3.48 cells, n = 10 and Met 59.45±4.01 cells, n = 11, ns), 5 h (control 53.83±3.38 cells, n = 11 and Met 59.42±4.43 cells, n = 12, ns), and 18 h (control 48.00±2.24 cells, n = 18 and Met 61.86±5.85 cells, n = 14, p<0.05) Only overnight Met treatment showed a significant increase in NR staining. **E)** Select images from a time-lapse video of 4 dpf *panther*;Ntr in 10 mM Met for 18 hrs. Puncta are seen along the axons at 5 h and 18 h (arrowheads). No other motile cells are seen surrounding the axons or ganglia. For all panels, anterior is to the left; dorsal is at the top. Eye and otic vesicle (OV) are outlined. Vagal ganglia labeled gX in panel E.

Previous work on myelinated axons has shown that macrophages are another class of phagocytic immune cell recruited in response to nerve injury [15], [18]. To determine if macrophages were recruited to degenerating axons and if they were a part of the population of Ntr/mCherry-labeled motile cells seen in the time-lapse videos, we tested Met treatment in larvae containing GFP-labeled macrophages (Ntr;mpeg1:gfp^gl22Tg^ ). Overnight treatment with 10 mM Met resulted in the appearance of mCherry-labeled motile cells in both the ganglia and nerve, and confocal imaging revealed that these were co-labeled with GFP (Figure 6A–A”). These findings demonstrated that macrophages had been recruited to the areas of cell and axonal degeneration and had actively phagocytosed labeled debris. To quantitate the numbers of recruited macrophages, Neutral Red (NR) staining was performed. NR is a vital stain that allows for selective identification of macrophages in larval zebrafish due to the large fused vacuolar aggregates present in these cells [48]. We incubated 4 dpf larvae in control PTU water or 10 mM Met for various times and carried out NR staining, with larvae imaged and NR-stained cells counted at 5 dpf (Figure 6B–D). An incubation time of 2, 3, or 5 hr in Met did not result in a significant increase in macrophages; however, there was a significant increase in macrophages after overnight treatment with Met (Figure 6D), suggesting that a majority of macrophages accumulated more than 5 hours after the start of Met treatment. It is important to note that the process of NR staining takes several hours in live larvae, so the indicated cell counts do not represent the number of macrophages present at the indicated incubation time, but instead reflect the number of NR-labeled macrophages that have accumulated as a result of the indicated incubation time in Met.

To verify that macrophages were recruited in response to axon degeneration, we examined Met-induced degeneration in a mutant with abnormal macrophage behavior, *panther*. In these mutants, a loss-of-function mutation in the MCSF-R (macrophage colony stimulating factor receptor) protein [40] causes a delay in the migration of macrophages out of the yolk sac and in their differentiation into microglia, resulting in a reduction of macrophages and microglia in the larval head until around 7 dpf [48]. When 4 dpf *panther*;Ntr larvae were treated with Met, time lapse imaging showed fluorescent puncta beginning to form at 2–3 hours along the axons and in the plexus (Movie S4; Figure 6E), similar to what was seen in wild type Ntr larvae (compare to Figure 1B’). However, the red motile cells seen in videos of Met-treated wild type Ntr failed to appear during an 18 hr period of Met exposure in the *panther*; Ntr larvae, suggesting that these red motile cells were primarily macrophages and/or microglia. To confirm these findings, we carried out NR staining on 4 dpf *panther*; Ntr larvae treated with 10 mM Met or control PTU water for various lengths of time: we did not detect a significant increase in macrophage numbers in response to Met exposure (data not shown). Together, the mpeg:gfp imaging, the NR staining and *panther*; Ntr data indicated that macrophage recruitment is one response to axon injury in this model.

### The typical inflammatory response to degeneration was dependent upon peripheral glia

**Figure 7:**
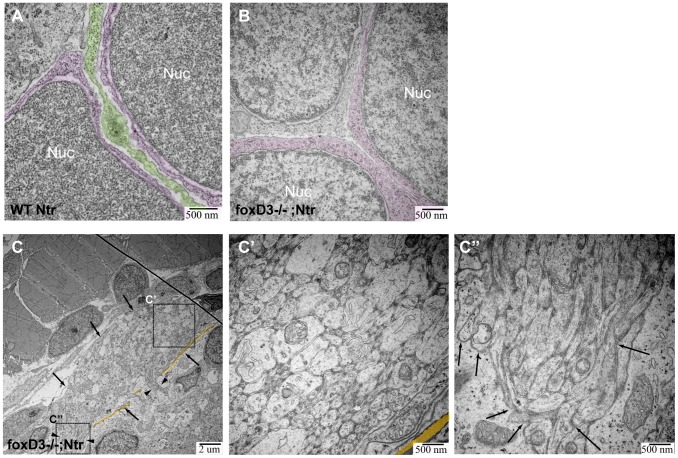
TEM images of missing peripheral glia in FoxD3^−/−^Ntr. **A)** Ganglion in 4 dpf wild type Ntr (40,000x) shows two cell bodies (nuclei, labeled Nuc; cytoplasm in magenta) with satellite glia (green) located in between. **B)** Ganglion in 4 dpf FoxD3^−/−^Ntr (30,000x) shows three adjacent cell bodies (nuclei in orange; cytoplasm in magenta) without satellite glia present. **C,C’,C’’)** Axons in 4 dpf FoxD3^−/−^;Ntr. C) Cross section of axon bundle (arrows) and longitudinal view of axons entering hindbrain (arrowheads) (7500x). Basement membrane indicated in orange in all images. C’) Higher magnification (30,000x) of axon bundle. Medium small axons appear relatively normal (compare to wild-type Ntr in Figure 4C’). C’’) Higher magnification (30,000x) shows defasciculated axons (arrows) entering brain (compare to Figure 4C’’).

In the absence of macrophages (*panther*;Ntr), Met-treatment still resulted in the appearance of puncta and the degenerating axons/neurons gradually lost their fluorescence (Figure 6E), indicating that debris from the axons and ganglia was still phagocytosed and degraded. This observation suggested that still another cell type was involved in the observed response. In the ganglia, likely candidates are the satellite glia, due to their proximity to neurons, while a probable cell type in the axons is the non-myelinating ensheathing glia. Previous work on myelinating Schwann cells in other animal models has shown that they phagocytose axonal debris and recruit macrophages in response to axon degeneration [17], [64]. We next wanted to test if the non-myelinating glia in the cranial nerves, the satellite and ensheathing glia, were responsible for the phagocytosis and the induced inflammatory response seen after neuronal degeneration. To accomplish this, we performed our next series of studies in a genetic background lacking peripheral glia. *FoxD3* is a transcription factor expressed during neural crest lineage specification that is proposed to be required for the generation of peripheral glia [39]. Using the *foxD3* ^zdf10^ mutant line [39], we generated *foxD3* ^+/−^; Ntr adults that allowed us to produce homozygous mutant larvae (readily detectable due to their stereotypical jaw defects) for our experiments. A caveat in the use of this mutant line is that the description of the *foxD3* phenotype is based on the absence of transcription factor mRNAs in peripheral glia [39], [65]. TEM thus provided us an opportunity to confirm that peripheral glia, both in the axon bundle and in the ganglia, were absent in these mutants, thereby providing confidence that any observed differences in Met-induced degeneration seen in the mutant larvae were due to the absence of peripheral glia. Satellite glia located between neuronal cell bodies were clearly observed in TEM analysis of the vagal ganglia in wild type Ntr (green, Figure 7A); however, these glia were not seen in any TEM images of *foxD3^−/−^*; Ntr (Figure 7B) ganglia. Figure 7C shows TEM images of a cross section of a vagal nerve bundle (arrows) along with a longitudinal view of axons entering the hindbrain (arrowheads) in an untreated *foxD3^−/−^;* Ntr larva The cross section of this nerve bundle appeared relatively normal when compared to wild type Ntr (compare Figure 7C’ with Figure 4C’); however, the axons entering the hindbrain (arrows) in untreated *foxD3^−/−^;*Ntr larvae were defasciculated and not parallel with each other (compare Figure 7C’’ with Figure 4C’’), which is in keeping with previous work from this lab showing partial defasciculation of the vagal nerve in *foxD3^−/−^* larvae [66]. We also performed TEM analysis on a non-Ntr expressing myelinated nerve, the posterior lateral line nerve, in mutant and wild type clutch mates to further confirm that these mutants do not express any peripheral glia. In 4 dpf wild type clutch mates, the axons are loosely wrapped by myelin and the cytoplasm of Schwann cells is seen in between axons. In 4 dpf *foxD3^−/−^* mutants, no myelin or glial cytoplasm is seen (Figure S4). This data confirms that peripheral glia are missing in these mutants.

**Figure 8:**
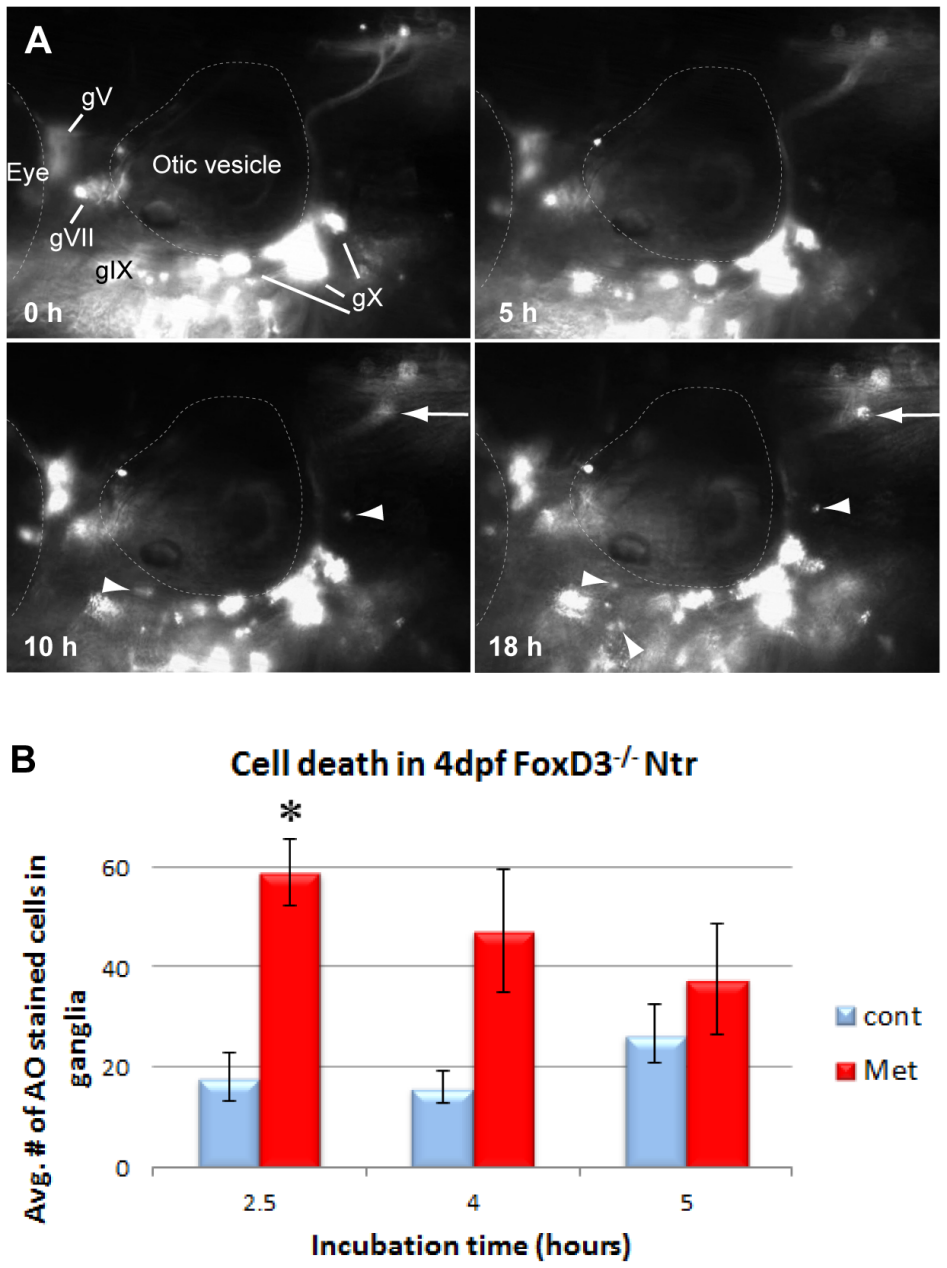
Inflammatory response in the absence of peripheral glia. **A)** Select images from a time lapse video of 4 dpf FoxD3^−/−^;Ntr treated with 10 mM Met for 18 h. Puncta around plexus (arrows) and red motile cells (arrowheads) appeared around 10 h after start of Met treatment. For comparison, wild type Ntr larvae developed puncta after 2–4 hr in Met (Figure 1B’). **B)** Quantitation of cell death in 4 dpf Met-treated FoxD3^−/−^Ntr compared to untreated FoxD3^−/−^;Ntr. AO-stained cell counts after various incubation times in 10 mM Met. There was a significant increase in cell death after 2.5 hour Met incubation (control 18.00±4.96, n = 9 and Met 59.06±6.66, n = 16, p<0.05), but not after 4 h (control 16.0±3.1 cells, n = 5 and Met 47.3±12.3, n = 6, ns) or 5 h (control 26.6±6.0 cells, n = 5 and Met 37.6±11.0, n = 5, ns) Met treatment. For all panels, anterior is to the left; dorsal is at the top. Eye and otic vesicle (OV) are outlined. Cranial ganglia labeled gV, gVII, gIX and gX in panel A.

Time lapse imaging of *foxD3^−/−^*; Ntr larvae treated with 10 mM Met (Movie S5; Figure 8A) revealed a substantial alteration in the pattern of debris phagocytosis when compared to treated wild-type clutch mates. The puncta normally observed along the peripheral nerve beginning around 2–4 hours post treatment were not detected, and the appearance of red motile cells consistently occurred with a marked delay (beginning at 9–10 hours in the mutants versus 2–4 hours seen in wild-type larvae) (see Figure 1B). Puncta were still observed within the CNS plexus, but the timing of their appearance was inconsistent: they could appear before, during or after the presence of red motile cells.

To determine if this delayed appearance of red motile cells and lack of puncta was perhaps due to postponed or decreased neuronal cell death in the mutants, AO staining was performed on 4 dpf *foxD3^−/−^;* Ntr incubated in 10 mM Met for 2.5 hr, 4 hr, or 5 hr, followed by counting of AO-stained cells in the ganglia (Figure 8B). Similar to wild type clutch mates, Met treatment in *foxD3^−/−^;* Ntr caused a significant increase in cell death during early stages of Met exposure (18.00±4.96, n = 9 in untreated and 59.06±6.66, n = 16 in 2.5 hour Met-treated larvae; p<0.001), indicating that the absence of peripheral glia was not interfering with Met-induced neuronal cell death. Indeed, it appeared that cell death in the mutants occurred to a greater degree, as there were a higher number of dead and dying cells in Met-treated mutants than in Met-treated wild type clutch mates.

**Figure 9:**
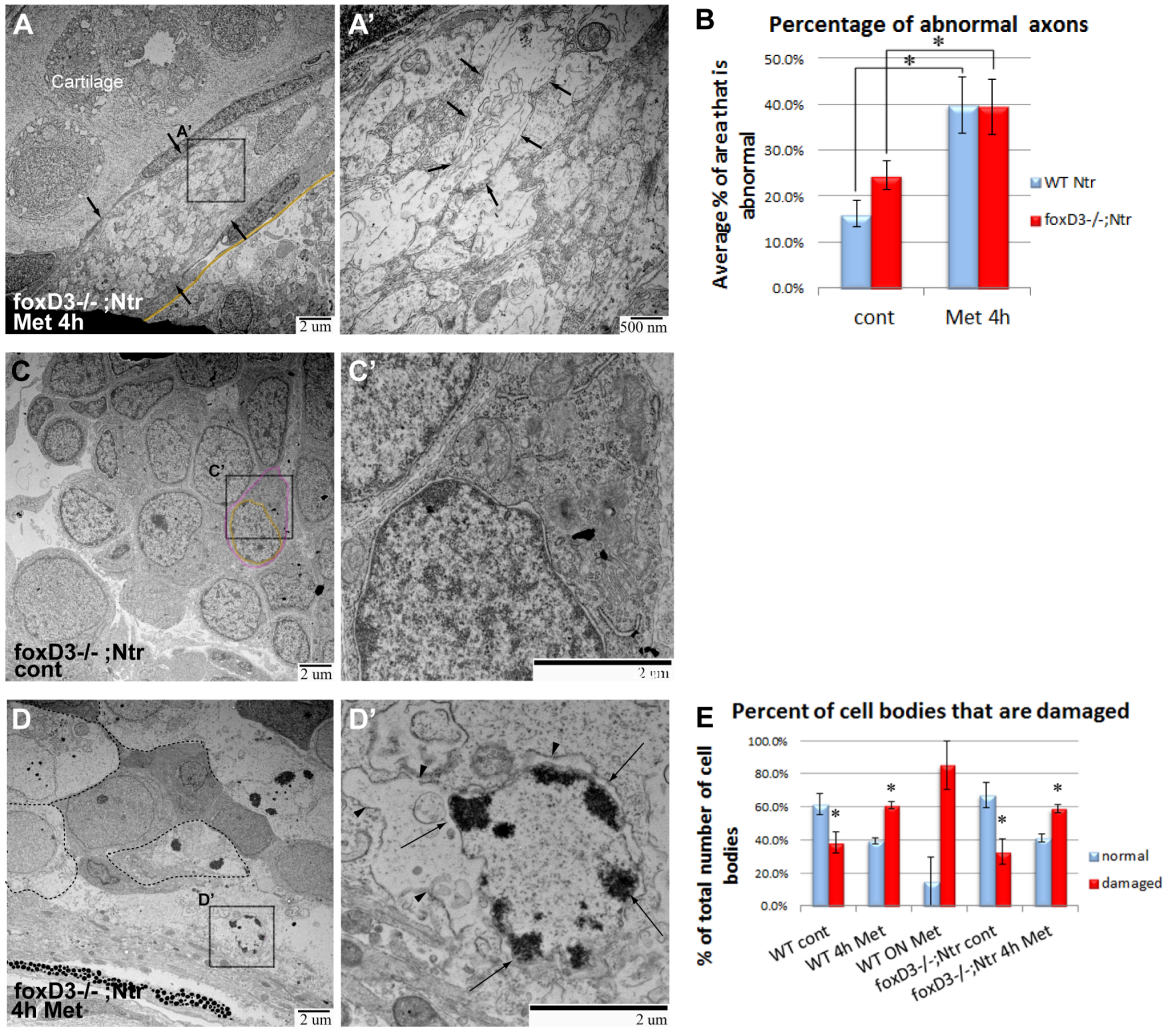
TEM images of axons and ganglia in 4^−/−^Ntr larvae. **A,A’)** Axons after 4) Cross section of axon bundle (arrows) (7500x). Membrane dividing brain and periphery (basement membrane) highlighted orange in all images. A’) Higher magnification (30,000x) of A. Individual axons (arrows) have abnormal appearance and broken membranes (compare to Figures 7C’ and 4D’). **B)** Quantitation of axon damage (given as percent of total axon area damaged) in Ntr and FoxD3^−/−^;Ntr. Damage in Ntr significantly increased from 16.2% (±2.84%, n = 10) in untreated to 39.9% (±6.08%, n = 5) with 4 hr Met treatment (p<0.01). In mutants, damage increased significantly from 24.53% (±3.19%, n = 7) in untreated to 39.48% (±5.97%, n = 3) in 4 hr Met-treated larvae (p<0.01). **C,C’)** Ganglia in untreated FoxD3^−/−^;Ntr (nucleus in orange; cytoplasm in magenta). C) Ganglia appear normal at low magnification (7500x) (nucleus in orange; cytoplasm in magenta; compare to Figure 5A). C’) Higher magnification (15,000x) shows normal cell body without surrounding glia. **D,D’)** Ganglia in FoxD3^−/−^;Ntr treated with 10 mM Met for 4 hr. D) Damaged cell bodies (outlined) are apparent at this magnification (7500x). D’) Higher magnification (15,000x) of degenerating cell. Nucleus has distinctive chromatin clumps (arrows). Cytoplasm contains damaged organelles and broken, irregular cell membranes (arrowheads, compare to Figure 5B’). **E)** Quantitation of Met-induced cell body damage in wild type Ntr and FoxD3^−/−^Ntr. Graph shows percent of normal versus damaged soma in wild type control (normal 61.7%±6.5%; damaged 38.3%±6.5%, n = 5, p<0.05), 4 h Met (normal 39.2%±2.0%; damaged 60.8%±2.0%, n = 5, p<0.05), 18 hr Met (normal 14.9%±14.9%; damaged 85.1%±14.9%, n = 2, ns), mutant control (normal 67.2%±7.8%; damaged 32.8%±7.8%, n = 2, p<0.05), and mutant 4 hr Met (normal 41.2%±2.5%; damaged 58.8%±2.5%, n = 3, p<0.05). Met treatment significantly increased the number of degenerating cells in wild type and mutant larvae after 4 hr Met treatment.

We next turned to TEM analysis to determine if Met-induced degeneration occurring in the absence of glia was different from that seen in wild-type Ntr larvae. 4 dpf *foxD3^−/−^;* Ntr larvae were treated with 10 mM Met for 4 hours or left untreated, then fixed and processed for TEM. In a nerve cross section of a 4 hour Met-treated *foxD3^−/−^;* Ntr larva (Figure 9A), there were several vacuole-like structures (arrows) that were consistent with a degeneration phenotype (Figure 9A’), however no dark puncta were apparent, as was seen in wild type larvae after a 4 hour Met treatment (Figure 4D’). The degeneration seen in these treated mutants more closely resembled what was seen in 18 hour, as opposed to 4 hour, Met-treated wild type larvae with regard to empty vacuoles and lack of dark puncta (compare Figure 9A’ to Figure 4E’). Quantitation of axon damage in *foxD3^−/−^;* Ntr larvae showed a significant increase in the percent of damaged axons in a nerve cross section with a 4 hour Met-treatment, similar to that seen for wild type larvae (Figure 9B). These data showed that the proportion of degenerating axons was similar in both wild type and mutant Ntr fish, and that axonal damage was more severe and occurred faster in the absence of peripheral glia.

The ganglia in untreated *foxD3^−/−^;* Ntr larvae showed intact neuronal cell bodies lying adjacent to each other without intervening glia (Figure 9C,C’). In 4-hour Met-treated *foxD3^−/−^;* Ntr larvae, degeneration and loss of membrane integrity in the ganglia were apparent at low magnifications (Figure 9D). Higher magnification showed degenerated cell bodies with condensed chromatin in nuclei (arrows), cell debris, and damaged membranes (arrowheads) (Figure 9D’). Again, these changes were more prominent than in corresponding 4 hour Met-treated wild type Ntr larvae (compare Figure 9D’ and Figure 5B’). Quantitation of damaged and abnormal cell bodies in the ganglia of *foxD3^−/−^;* Ntr larvae showed a significant increase in the number of damaged somas with Met treatment (32.8%±7.8%, n = 2 in untreated and 58.8%±2.5%, n = 3 in Met-treated larvae, p<0.05) (Figure 9E). These findings suggested that similar to what occurred in the axons, without satellite glia Met treatment caused accelerated and more severe degeneration in the somas.

From our TEM analysis, we found that mutant larvae had damaged somas and axons with poor membrane integrity after a 4 hour Met treatment; however, in time-lapse videos of these mutants, engulfment of cell debris did not appear until 8–10 hours into Met treatment. In Met-treated wild type clutch mates, damage was apparent by 4 hours and this was concurrent with the appearance of phagocytic cells. These findings suggest that satellite and ensheathing glia are necessary for debris phagocytosis.

The second response typically seen in the wild type Met-treated clutch mates is the appearance of red motile cells in and around the nerve and the ganglia. To determine if there was a normal recruitment of immune cells in the absence of peripheral glia, we stained for both neutrophils and macrophages. There was no significant difference in the number of SB stained cells between untreated (38.5±8.6 cells/fish, n = 8) and 4 hr-Met treated mutants (33.9±7.9 cells/fish, n = 10) (data not shown), indicating that the number of neutrophils did not increase. We also carried out NR staining on *foxD3^−/−^;* Ntr larvae to examine macrophage recruitment, and again there was no significant difference in NR stained cells after 2.5 hr between untreated (30.3±3.2 cells/fish, n = 7) and Met treated mutants (22.3±1.0 cells/fish, n = 4), or 18 hr between untreated (37.2±8.6 cells/fish, n = 5) or Met treated mutants (43.5±8.6 cells/fish, n = 6). These findings were surprising given the fact that time lapse videos of Met-treated *foxD3^−/−^;* Ntr showed that motile red cells were eventually recruited (Figure 8A). This delay could be due to either the lack of peripheral glia or to an alteration in the immune response in the mutants. We ruled out the latter possibility by demonstrating a normal neutrophil and macrophage response to injury using the tail transection assay [67] (Figure S5). Therefore, the simplest explanation for the non-significant change in macrophage numbers is that lower numbers of macrophages were recruited in the mutants. Overall, these data suggest that immune cell recruitment to degenerating axons and cell bodies was impaired by the absence of peripheral glia.

## Discussion

Degeneration is a hallmark feature of most PNs. These disorders have a variety of causes and symptoms, affect millions of people, and have no cures. Insults leading to a PN can arise in any type of nerve, can include both myelinated and unmyelinated axons, and often result in axon degeneration and death of the neuron [55]. A common animal model for axon degeneration involves peripheral nerve transection, a physical trauma that results in damage not only to axons but to the surrounding non-neural tissue as well. Oftentimes the nerves targeted in such models contain myelinated, or a combination of myelinated and unmyelinated, axons. While much is known about the response to degeneration of myelinated axons, little is known concerning the organismal response to degeneration of the small unmyelinated fibers. This is an important deficit as many painful PNs, such as those caused by diabetes [14], involve this class of axons.

To investigate unmyelinated axon degeneration, we generated a novel zebrafish model. The advantages of this model are that degeneration is targeted specifically to the peripheral sensory neurons, it is conditional, it can be visualized in vivo, the affected nerves are unmyelinated, and many aspects of the organismal response to this degeneration mimic what is seen in the clinical setting. We also demonstrate that the initial response to neuron degeneration is dependent upon the peripheral non-myelinating glia that ensheath both the axons and the cell bodies.

### Met-induced neurodegeneration recapitulates that seen in peripheral neuropathies

While the initial insults that lead to PN in humans are varied, common outcomes include cell death, DNA damage, disruption of microtubules, defects in proteins required for fission and fusion of mitochondria, high levels of reactive oxygen species (ROS) causing oxidative stress, and induction of an inflammatory response [65]–[67]. In this report, we establish that our model can be used to selectively kill peripheral sensory neurons, induce axon degeneration, and elicit an inflammatory response. This process can be visualized in real time in a live organism and permits us to see axon degeneration in an appropriate context with all cell types present.

Neuronal degeneration and death via apoptosis in human PNs is linked to an increase in neuronal ROS concentrations and mitochondrial dysfunction [55], [68], [69]. Nitroreductases, such as the one expressed in our zebrafish model, have been shown to convert the prodrug Met into free radical metabolites (including ROS) [33], which are cytotoxic and result in the death of the cells expressing the enzyme– in our case, the peripheral sensory neurons of the zebrafish. The features of Met-induced axon degeneration observed in our model are reminiscent of those seen in many PNs caused by drugs, toxins, or metabolic insults: electron micrographs revealed that the amount of membrane disruption within an axon bundle was increased in damaged nerves, microtubule densities within individual axons appeared to be reduced, and axonal mitochondria lost structural definition.

Vulnerability of select axons is seen in many human PNs, where the sensory neurons are affected while the motor neurons intact remain intact; *e.g.,* chemotherapy-induced sensory PNs [2], toxic insults (e.g. pyridoxine toxicity [9]), and hereditary sensory neuropathies [7]. We were able to replicate this selectivity by expressing the Ntr-mCherry chimeric protein within sensory neurons and demonstrated there was no detectable bystander effect on adjacent motor axons upon Met treatment. This ability of the Ntr/Met system to selectively ablate targeted cell types within a tissue has also been shown for beta cells in the pancreas [28] and podocytes in the kidney [27].

Met treatment resulted in neuronal death slightly before, or contemporaneously with, axonal degeneration. Axon damage first appeared as a loss of definition of individual axons within a nerve, followed by the appearance of puncta (phagocytosed debris) and recruitment of immune cells. This is similar to what has been reported for degeneration in other animal models of PN. However, we did not observe a significant increase in the number of neutrophils responding to axon degeneration, which is in contrast to studies in rats and mice that have shown significant neutrophil recruitment in dorsal root ganglia after sciatic chronic constriction injury [60], [70] and in zebrafish larvae after tail transection [71], [72]. There are two possibilities for this difference: selective axon degeneration initiated by a drug may not produce the same cytokines to recruit neutrophils as the non-specific trauma induced by nerve transection, or that it is the myelin proteins that are a key element in neutrophil recruitment. Support for the idea that there is a different repertoire of cell signals produced from damaged unmyelinated versus myelinated nerves comes from earlier studies investigating destruction of unmyelinated primary afferent nerves in rat using capsaicin. Those experiments also showed a reduced neutrophil response [73], [74].

Neutrophils are not the only immune cell that can be recruited to damaged or dying axon/neurons. Our data showed that macrophages were recruited to degenerating axons, which agrees with the well-established characteristics of Wallerian degeneration and the recruitment of macrophages in models using nerve transection, ligation, and crush [15], [18]. However, macrophages in our drug-induced sensory axon degeneration model appear several hours later than macrophages in a motor axon axotomy model in zebrafish [75]. Potential reasons for this variance in timing include differences in 1) sensory versus motor axon degeneration; 2) drug-induced axon degeneration versus axotomy-induced Wallerian degeneration; or 3) myelinated versus non-myelinated axons. Since others have shown that myelin has an important role in macrophage recruitment [18], the lack of myelin in our axon degeneration model is a strong possibility for the delay in the appearance of macrophages. In videos of Met-treated mutants lacking macrophages, puncta are seen in the axons, fluorescence in the ganglia gradually disappears, and there is apparent cell movement in the ganglia, similar to Met-treated wild type Ntr. These findings indicate that an additional cell population is present and responsible for phagocytosing debris. The most likely candidates for these phagocytic cells are the non-myelinating glia of the ganglia (satellite cells) and axons (ensheathing glia).

### Peripheral glia are required for the normal response to axonal degeneration

When Ntr^+^ mutants lacking peripheral glia (*foxd3* ^−/−^) were exposed to Met, no discernible puncta were observed along the axons during treatment, and the appearance of immune cells was dramatically delayed. This was not due to decreased degeneration of sensory neurons/axons, and in fact the opposite occurred: axon degeneration advanced more rapidly in the *foxD3^−/−^;*Ntr than in wild type. TEM analysis revealed that axons in mutants treated with Met for only 4 hours contained the large vacuoles and broken membranes that more closely resembled axons from wild types treated overnight with Met. Also, dark puncta located in the axons of Met-treated wild type larvae were not seen in mutants, suggesting that these inclusions were formed due to the presence of peripheral glia. In the ganglia of mutants exposed to Met for 4 hours, degenerating cell bodies had more apparent membrane damage and nuclear defects than equivalently-treated wild type larvae.

While puncta were not observed in the axons of treated *foxd3^−/−^* larvae, recruitment of immune cells still occurred, although considerably delayed. In wild type larvae, immune cells were recruited starting at 4–5 hours of Met treatment. In the mutants, this initial response has been delayed to more than 8–10 hours of Met exposure. In addition, the response in the mutants is less robust than that seen in their wild-type clutch mates. Since the red motile cells seen in both *foxd3^−/−^* mutants and wild types had similar sizes and movements, such as the speed they migrated and the extension of philopodia, it is assumed that they are macrophages and/or microglia. In addition, no phagocytic cells were detected in TEM images in the ganglia of Met-treated mutants, as was seen in Met-treated wild types. This suggests that these non-myelinating peripheral glial cells have a pivotal role in immune cell recruitment.

These findings are supported by previous studies demonstrating that non-myelinating glia express cytokines and chemokines that are known to recruit immune cells in a capsaicin-induced degeneration model [76], [77]. In contrast to these studies, Rosenberg et al. showed that macrophage recruitment is independent of myelinating Schwann cells after laser axotomy of motor axons in *sox10^−/−^* zebrafish mutants that lack those glia [75]. These findings underscore the need to investigate the cellular mechanisms underlying responses of different glial subtypes to different types of insults/injuries.

### Unmyelinated versus myelinated axon degeneration

One of the major differences between our study and those carried out in mammals is that the nerves we targeted contain only unmyelinated axons. Studies have shown that myelin plays a significant role in immune cell recruitment and in hindering regeneration of axons. Degrading myelin expresses complement C3, which is important in opsonization and phagocytosis of myelin by macrophages [18]. Degraded myelin also activates NF-κB and c-Jun through toll-like receptors (TLRs), increasing expression of inflammatory cytokine genes in macrophages [78]. These studies highlight the critical role of myelin in immune cell recruitment; however, immune cells still respond to unmyelinated axon degeneration. This stresses the need for a better understanding of the inflammatory response in the absence of myelin. There is evidence that myelinated and unmyelinated axons have different cytokine and chemokine expression profiles during Wallerian degeneration. For instance, degenerating unmyelinated axons did not increase mRNA expression of IL-1β and LIF (leukemia-inhibitory factor), had lower levels of MCP-1 (monocyte chemotactic protein-1), IL-2, and IL-10 when compared to transected nerves, and peak expression levels of some cytokines occurred earlier in degenerated unmyelinated axons compared to whole transected nerves [77]. Understanding differences between myelinated and unmyelinated axon degeneration is important in developing therapeutic targets specific for different types of PNs, such as small fiber neuropathies, where only small unmyelinated axons are affected [21].

In this paper, we describe a new model to investigate drug-induced peripheral sensory axon degeneration and hypothesized that non-myelinating glia have a crucial role in the inflammatory response to axon degeneration and phagocytosis of debris. We approached this using the Ntr/Met system to ablate peripheral sensory neurons and assess the inflammatory response, cell death, and the ultrastructure of degenerating axons and cell bodies in both wild type and mutant larvae lacking peripheral glia. Using this method, we found that administering Met caused axon degeneration and neuronal cell death in Ntr-expressing cells, and also elicited an inflammatory response. Using the *foxD3^−/−^;* Ntr mutant that lacks peripheral glia, we highlight the importance of non-myelinating satellite and ensheathing glia in initiating the inflammatory response and in phagocytosing cell debris. Without satellite and ensheathing glia, neutrophil and macrophage recruitment is postponed, causing a delay in the clearance of harmful debris. This debris can potentially prevent successful regeneration and functional recovery of affected neurons.

Some of our findings involving the inflammatory response differed from other studies, such as the lack of significant neutrophil contribution [72], timing of macrophage appearance, and dependence of macrophages on Schwann cells for recruitment [75]. This suggests that there are differences in the inflammatory response between transected axons and drug-induced axon degeneration, myelinated and unmyelinated axons, and sensory versus motor axon degeneration. In this report, we describe a new animal model for axon degeneration, which implicated non-myelinating glia as a crucial player in this process. Future studies using this model will be fundamental for developing therapeutics for specific PNs, especially those involving unmyelinated peripheral sensory neurons, such as painful small fiber neuropathies.

## Supporting Information

Figure S1 **Complete ablation of Ntr-expressing peripheral sensory neurons after Met treatment.** 4 dpf Ntr larvae treated with A) PTU water (control) or B) 10 mM Met for 18 hours. Confocal microscopy at 5 dpf revealed that vagal nerve axons (A, arrowheads) remain intact in control animals, but lose all structural integrity when treated overnight with Met (B). Anterior to left, dorsal at top.
(TIF)

Figure S2 **Met treatment increases mitochondrial damage in axons.** TEM images show examples of healthy (A) and unhealthy (B) mitochondria (arrowheads) present in EM sections of control or treated vagal nerve bundles. Mitochondria were scored and the percentage of mitochondria found as healthy or unhealthy is shown in panel C (number of mitochondria evaluated: untreated controls, n = 82; 4 hr treatment n = 52, 18 hr treatment n = 88, number of fish examined per group ranged from 3–6).
(TIF)

Figure S3 **Neutrophil response to degenerating neurons in 4** **dpf Ntr larvae. A)** Sudan Black staining of Ntr treated with fish water (control) or 10 mM Met for 4 hr, (neutrophils labeled with arrowheads). **B)** SB-stained cell counts in larvae treated with control or 10 mM Met for various times. Average number of SB-stained cells/fish per treatment group shown for incubation times of 3 h (control 25.31±3.81, n = 16; Met 26.00±4.19, n = 17), 4 h (control 20.47±3.55, n = 17; Met 28.12±3.49, n = 17), 5 h (control 18.67±4.84, n = 6; Met 28.17±6.79, n = 6), 6 h (control 21.67±4.36, n = 6; Met 31.17±6.06, n = 6), and 18 h (control 21.55±2.55, n = 11; Met 23.92±2.95, n = 12). None were statistically significant. **C)** Neutrophil movement in Mpx:gfp;Ntr larvae treated with fish water or 10 mM Met. Graph shows average velocity (pixels/sec) of neutrophils over time: 0–0.5 h (control 0.165±0.008, n = 44; Met 0.152±0.017, n = 42, ns), 0.5–1 h (control 0.149±0.018, n = 15; Met 0.144±0.013, n = 32, ns), 1–1.5 h (control 0.112±0.007, n = 19; Met 0.075±0.023, n = 4, ns), 1.5–2 h (control 0.111±0.010, n = 17; Met 0.192±0.012, n = 93, p<0.05), 2–2.5 h (control 0.110±0.007, n = 51; Met 0.164±0.006, n = 64, p<0.05), 2.5–3 h (control 0.141±0.017, n = 23; Met 0.147±0.009, n = 50, ns), 3–3.5 h (control 0.137±0.011, n = 24; Met 0.207±0.032, n = 53, ns), 3.5–4 h (control 0.122±0.005, n = 51; Met 0.123±0.013, n = 24, ns), 4–4.5 h (control 0.124±0.008, n = 51; Met 0.253±0.027, n = 91, p<0.05), 4.5–5 h (control 0.126±0.006, n = 44; Met 0.187±0.017, n = 33, p<0.05), 5–5.5 h (control 0.113±0.014, n = 24; Met 0.183±0.010, n = 60, p<0.05), and 5.5–6 h (control 0.129±0.017, n = 30; Met 0.206±0.015, n = 36, p<0.05). Anterior is to the left; dorsal is at the top in all panels. Eye and otic vesicle (OV) are outlined.
(TIF)

Figure S4 **TEM images of posterior lateral line nerve in wild type and** ***foxD3^−/−^;*** **Ntr larvae. A)** Cross section of the posterior lateral line nerve (pLL) in 4 dpf wild type Ntr (15,000x) shows axons surrounded by myelin (arrowheads). **B)** pLL in 4 dpf *foxD3^−/−^;*Ntr (15,000x) shows axons with adjacent axonal membranes (arrowheads). No myelin is seen.
(TIF)

Figure S5 **Loss of foxD3 does not result in an impaired immune response.** Tail transections were performed on 4 dpf *foxD3^−/−^;mpx*:gfp or WT clutch mates. Larvae were anesthetized with tricaine and tails were transected with a scalpel at the junction of the body and tail fin. 2 hr after the tail transection, larvae were either imaged for GFP+ cells (mpx:gfp, neutrophils) or stained with NR to visualize macrophages. **A)** Uncut tail of WT clutch mate shows the location of the tail transection (dashed line in all images). **A’)** Transection site of WT clutch mate showed an increase in neutrophils at the location of the cut. **B)** Uncut tail of *foxD3^−/−^;mpx*:gfp shows location of tail transection. **B’)** Transected tail of *foxD3^−/−^;mpx*:gfp also showed an increase in neutrophils at the location of the cut (arrow), similar to that seen in WT clutch mates. **C)** Neutral Red staining in uncut tail of WT clutch mate. **C’)** NR staining at transection of WT clutch mate (arrow) showed an increase in macrophages at the location of the cut. **D)** NR staining at the transection site in a *foxD3^−/−^;mpx*:gfp larva. **D’)** NR staining of transection site in a *foxD3^−/−^;mpx*:gfp showed an increase in macrophages at the location of the transection (arrow), similar to that seen in WT clutch mates.
(TIF)

Movie S1 **Timelapse video of Met-treated Ntr larva.** 4 dpf Ntr larva treated with 10 mM Met for 18 hours. Images taken every 12 min. Anterior to left, dorsal at top.
(MP4)

Movie S2 **Timelapse video of untreated** ***mpx:gfp*** **;Ntr larva.** 4 dpf *mpx:gfp*;Ntr treated with fish water. Images taken every 30 sec. Video spans a time frame of 30 min taken 4 hours after addition of fish water. Anterior to left, dorsal at top.
(MP4)

Movie S3 **Timelapse video of Met-treated** ***mpx:gfp*** **;Ntr larva.** 4 dpf *mpx;gfp*;Ntr treated with 10 mM Met. Images taken every 30 sec. Video spans a time frame of 30 min taken 1.5 hours after the start of Met treatment. Anterior to left, dorsal at top.
(MP4)

Movie S4 **Timelapse video of Met-treated** ***panther*** **;Ntr larva.** 4 dpf *panther*;Ntr larva treated with 10 mM Met for 18 hours. Images taken every 5 min. Anterior to left, dorsal at top.
(MP4)

Movie S5 **Timelapse video of Met-treated** ***foxD3^−/−^;*** **Ntr larva.** 4 dpf *foxD3^−/−^;*Ntr larva treated with 10 mM Met for 23 hours. Images taken every 5 min. Anterior to left, dorsal at top.
(MP4)
