# Supplementary figures and images for: Peripheral Glia Have a Pivotal Role in the Initial Response to Axon Degeneration of Peripheral Sensory Neurons in Zebrafish
Source: PLoS One. 2014 Jul 24;9(7):e103283. doi: 10.1371/journal.pone.0103283 (PMC4109997; doi:10.1371/journal.pone.0103283)

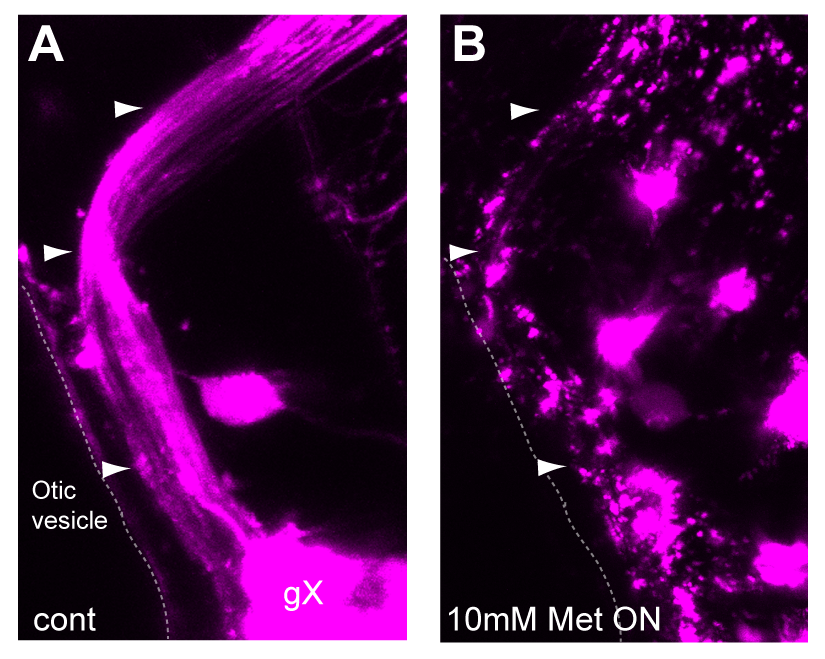

Supplement: Figure S1 — Complete ablation of Ntr-expressing peripheral sensory neurons after Met treatment. 4 dpf Ntr larvae treated with A) PTU water (control) or B) 10 mM Met for 18 hours. Confocal microscopy at 5 dpf revealed that vagal nerve axons (A, arrowheads) remain intact in control animals, but lose all structural integrity when treated overnight with Met (B). Anterior to left, dorsal at top. (TIF) [file pone.0103283.s001.tif]

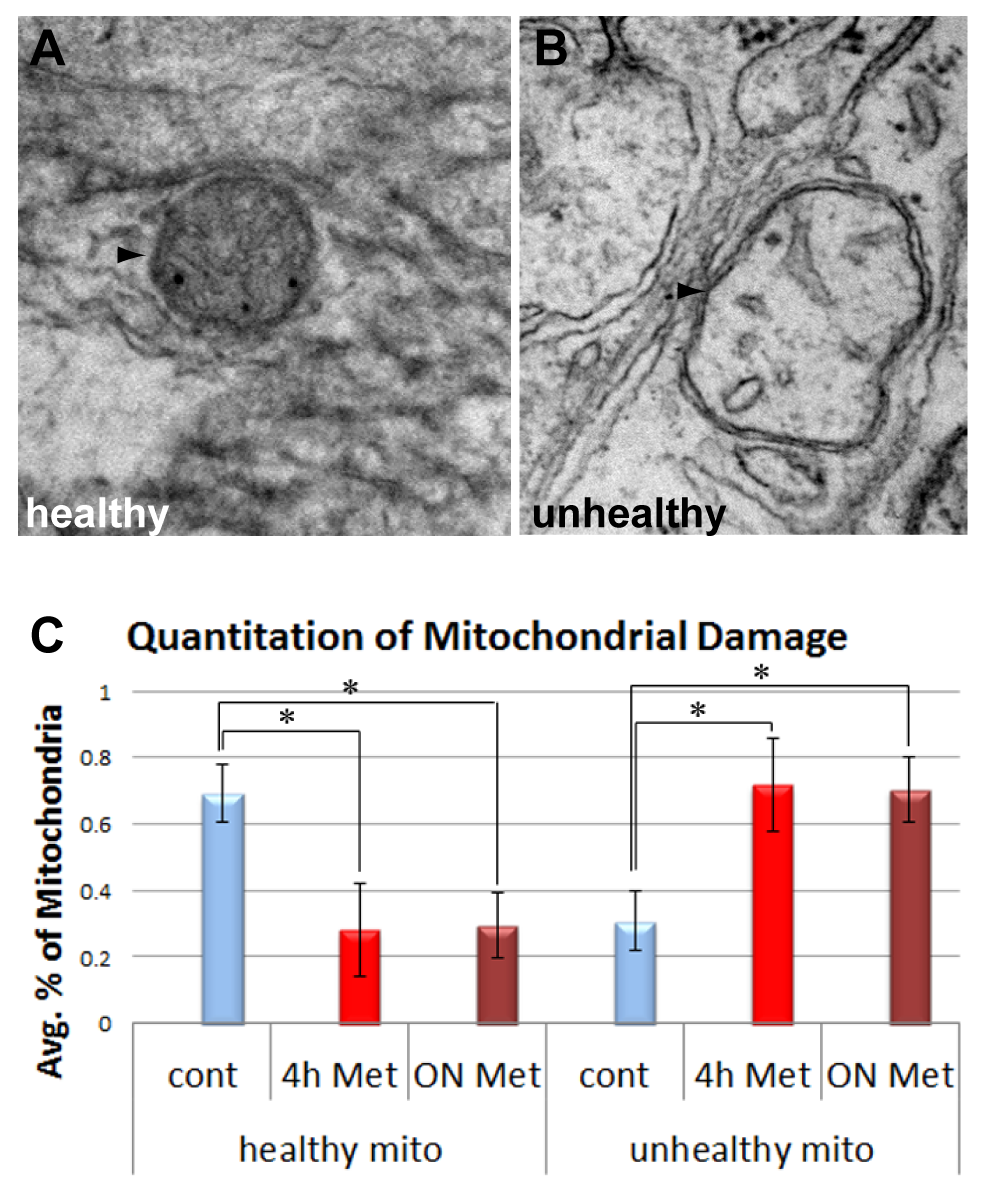

Supplement: Figure S2 — Met treatment increases mitochondrial damage in axons. TEM images show examples of healthy (A) and unhealthy (B) mitochondria (arrowheads) present in EM sections of control or treated vagal nerve bundles. Mitochondria were scored and the percentage of mitochondria found as healthy or unhealthy is shown in panel C (number of mitochondria evaluated: untreated controls, n = 82; 4 hr treatment n = 52, 18 hr treatment n = 88, number of fish examined per group ranged from 3–6). (TIF) [file pone.0103283.s002.tif]

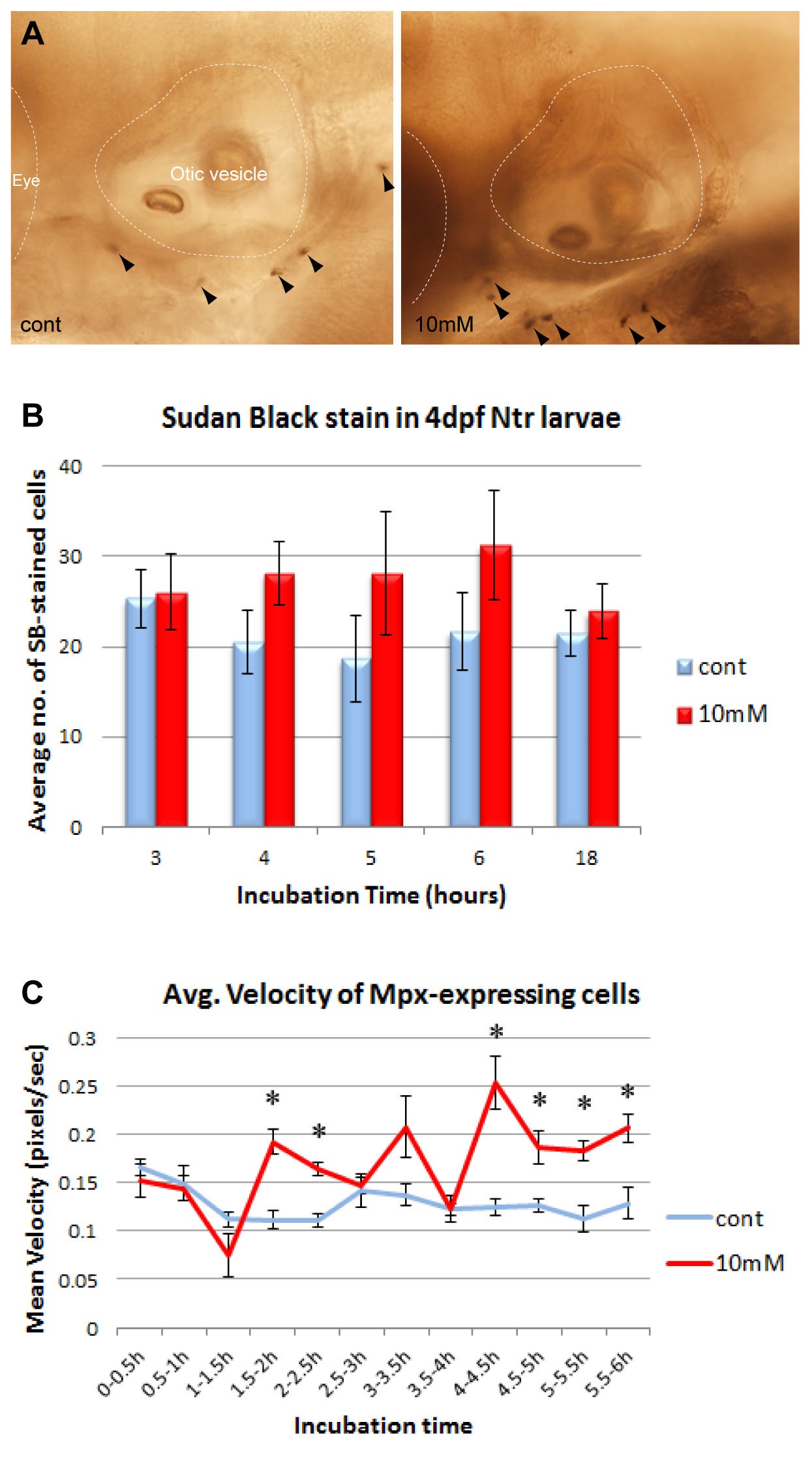

Supplement: Figure S3 — Neutrophil response to degenerating neurons in 4 dpf Ntr larvae. A) Sudan Black staining of Ntr treated with fish water (control) or 10 mM Met for 4 hr, (neutrophils labeled with arrowheads). B) SB-stained cell counts in larvae treated with control or 10 mM Met for various times. Average number of SB-stained cells/fish per treatment group shown for incubation times of 3 h (control 25.31±3.81, n = 16; Met 26.00±4.19, n = 17), 4 h (control 20.47±3.55, n = 17; Met 28.12±3.49, n = 17), 5 h (control 18.67±4.84, n = 6; Met 28.17±6.79, n = 6), 6 h (control 21.67±4.36, n = 6; Met 31.17±6.06, n = 6), and 18 h (control 21.55±2.55, n = 11; Met 23.92±2.95, n = 12). None were statistically significant. C) Neutrophil movement in Mpx:gfp;Ntr larvae treated with fish water or 10 mM Met. Graph shows average velocity (pixels/sec) of neutrophils over time: 0–0.5 h (control 0.165±0.008, n = 44; Met 0.152±0.017, n = 42, ns), 0.5–1 h (control 0.149±0.018, n = 15; Met 0.144±0.013, n = 32, ns), 1–1.5 h (control 0.112±0.007, n = 19; Met 0.075±0.023, n = 4, ns), 1.5–2 h (control 0.111±0.010, n = 17; Met 0.192±0.012, n = 93, p<0.05), 2–2.5 h (control 0.110±0.007, n = 51; Met 0.164±0.006, n = 64, p<0.05), 2.5–3 h (control 0.141±0.017, n = 23; Met 0.147±0.009, n = 50, ns), 3–3.5 h (control 0.137±0.011, n = 24; Met 0.207±0.032, n = 53, ns), 3.5–4 h (control 0.122±0.005, n = 51; Met 0.123±0.013, n = 24, ns), 4–4.5 h (control 0.124±0.008, n = 51; Met 0.253±0.027, n = 91, p<0.05), 4.5–5 h (control 0.126±0.006, n = 44; Met 0.187±0.017, n = 33, p<0.05), 5–5.5 h (control 0.113±0.014, n = 24; Met 0.183±0.010, n = 60, p<0.05), and 5.5–6 h (control 0.129±0.017, n = 30; Met 0.206±0.015, n = 36, p<0.05). Anterior is to the left; dorsal is at the top in all panels. Eye and otic vesicle (OV) are outlined. (TIF) [file pone.0103283.s003.tif]

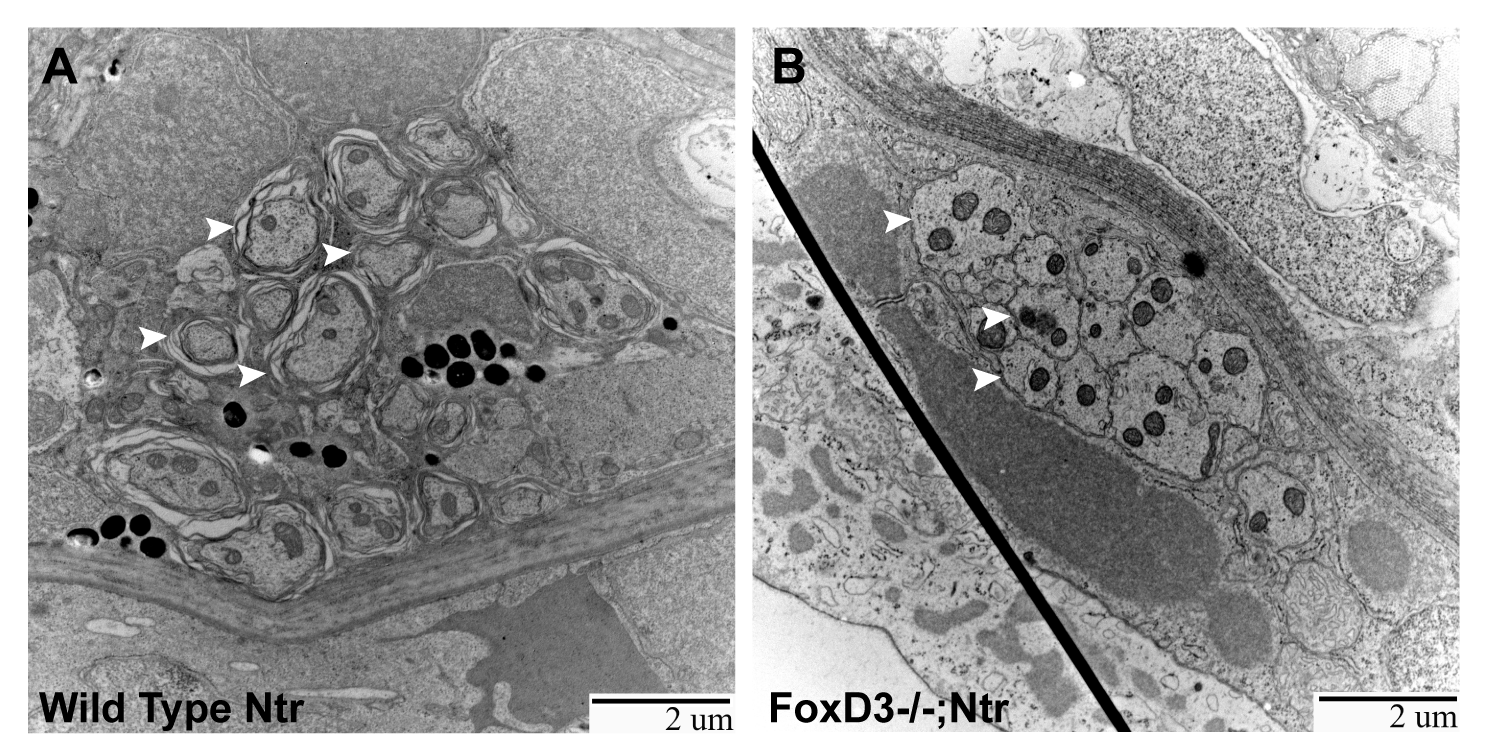

Supplement: Figure S4 — TEM images of posterior lateral line nerve in wild type and foxD3−/−; Ntr larvae. A) Cross section of the posterior lateral line nerve (pLL) in 4 dpf wild type Ntr (15,000x) shows axons surrounded by myelin (arrowheads). B) pLL in 4 dpf foxD3−/−;Ntr (15,000x) shows axons with adjacent axonal membranes (arrowheads). No myelin is seen. (TIF) [file pone.0103283.s004.tif]

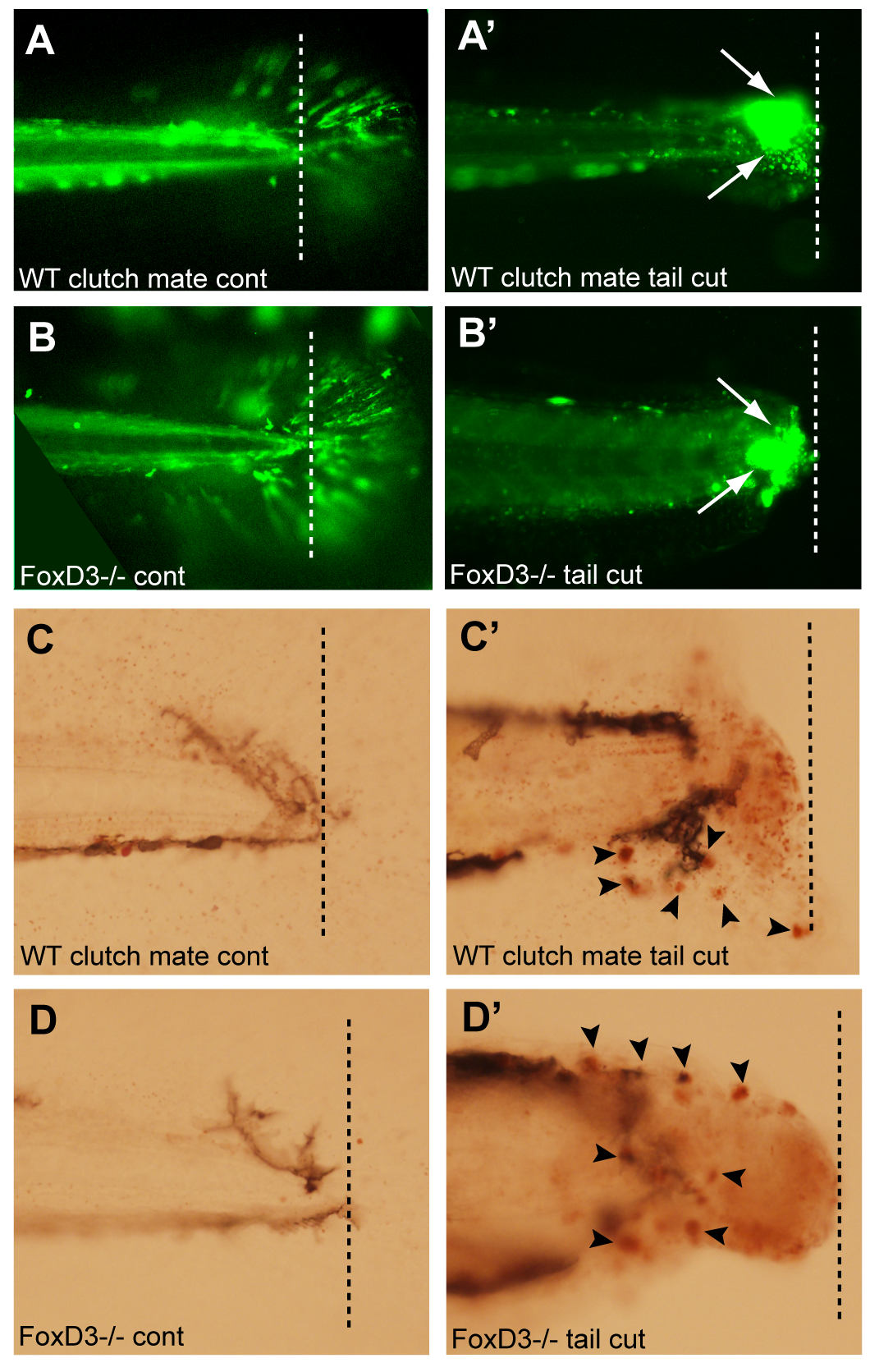

Supplement: Figure S5 — Loss of foxD3 does not result in an impaired immune response. Tail transections were performed on 4 dpf foxD3−/−;mpx:gfp or WT clutch mates. Larvae were anesthetized with tricaine and tails were transected with a scalpel at the junction of the body and tail fin. 2 hr after the tail transection, larvae were either imaged for GFP+ cells (mpx:gfp, neutrophils) or stained with NR to visualize macrophages. A) Uncut tail of WT clutch mate shows the location of the tail transection (dashed line in all images). A’) Transection site of WT clutch mate showed an increase in neutrophils at the location of the cut. B) Uncut tail of foxD3−/−;mpx:gfp shows location of tail transection. B’) Transected tail of foxD3−/−;mpx:gfp also showed an increase in neutrophils at the location of the cut (arrow), similar to that seen in WT clutch mates. C) Neutral Red staining in uncut tail of WT clutch mate. C’) NR staining at transection of WT clutch mate (arrow) showed an increase in macrophages at the location of the cut. D) NR staining at the transection site in a foxD3−/−;mpx:gfp larva. D’) NR staining of transection site in a foxD3−/−;mpx:gfp showed an increase in macrophages at the location of the transection (arrow), similar to that seen in WT clutch mates. (TIF) [file pone.0103283.s005.tif]
